# The nasty neighbor effect in humans

**DOI:** 10.1126/sciadv.adm7968

**Published:** 2024-06-26

**Authors:** Angelo Romano, Jörg Gross, Carsten K. W. De Dreu

**Affiliations:** ^1^ Social, Economic and Organisational Psychology Department, Leiden University, Leiden, Netherlands.; ^2^ Department of Psychology, University of Zurich, Zurich, Switzerland.; ^3^ Faculty of Behavioural and Social Science, University of Groningen, Groningen, Netherlands.; ^4^ Behavioral Ecology and Sociobiology Unit, German Primate Center, Leibniz Institute for Primate Research, Göttingen, Germany.

## Abstract

Like other group-living species, humans often cooperate more with an in-group member than with out-group members and strangers. Greater in-group favoritism should imply that people also compete less with in-group members than with out-group members and strangers. However, in situations where people could invest to take other’s resources and invest to protect against exploitation, we observed the opposite. Akin to what in other species is known as the “nasty neighbor effect,” people invested more when facing an in-group rather than out-group member or stranger across 51 nations, in different communities in Kenya, and in representative samples from the United Kingdom. This “nasty neighbor” behavior is independent of in-group favoritism in trust and emerges when people perceive within-group resource scarcity. We discuss how to reconcile that humans exhibit nastiness and favoritism toward in-group members with existing theory on in-group favoritism.

## Teaser

Humans not only cooperate but also compete more with in-group members than with out-group members and strangers.

## INTRODUCTION

Across the behavioral and biological sciences, there is ample evidence that people treat members of one’s language, political, or national group more favorably (*1*–*4*). This behavioral tendency, reminiscent of in-group favoritism (*2*, *5*–*17*) and sometimes referred to as “in-group love” (*10*) or parochialism (*12*, *14*, *15*, *18*–*20*), is widespread around the world (*11*, *12*, *21*). From this observation and extant literature, it may be inferred that people also compete more readily with outsiders than with insiders. If true, then this could explain why conflict often is more likely and intense between rather than within groups (*5*, *6*, *19*, *20*, *22*, *23*).

Existing evidence for in-group member favoritism is, however, largely based on measures of within- and between-group cooperation (*24*) or measures that combine cooperation and competition (*5*, *16*, *17*). In these situations, people can extend a benefit to others at some cost to themselves. In-group favoritism implies that people more readily extend benefits and create joint welfare when interacting with an in-group (rather than out-group) member. It should, therefore, follow that people also expend less resources when interacting with in-group (rather than out-group) members when this imposes a cost on others at a benefit to oneself (i.e., competition).

Psychologically, lack of cooperation—extending a benefit to others at some cost to oneself—cannot, however, be equated with the presence of competition—imposing a cost on others to benefit oneself (*25*). For example, it has been observed that people are often more cooperative but can likewise be more competitive than would be expected under rational choice theory (*26*–*28*). By implication, evidence for in-group favoritism in cooperation cannot be taken as evidence for lower competition toward in-group members, also known as out-group derogation (*2*, *19*, *26*, *29*). The few studies that considered favoritism in both cooperation and competition revealed mixed results, with some reporting that people compete more with distant others and out-group members (*6*, *16*) and others reporting no or even counterevidence (*26*, *30*–*33*) [for an overview, see (*2*, *3*, *33*)].

**Fig. 1.**
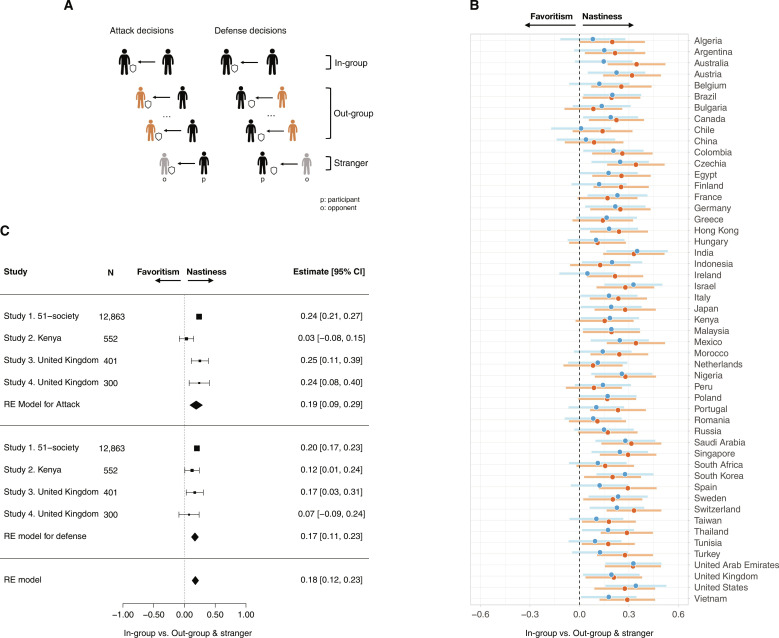
The nasty neighbor effect in humans. (**A**) Design for studies 1 to 4. Participants (p) invested resources in the AD-C across two randomized blocks. In one block (shown on the left), participants could invest their money to take money from an opponent (sampled from the pool of countries participating in the study). In the other block (shown on the right), they could invest to defend themselves against their antagonist’ attempt to take their money. In each block, they made one decision with an opponent from the same nation, 25 (in study 1; 16 in studies 3 and 4) decisions with foreigners from different nations and one decision with an unidentified stranger (in random order). (**B**) Conflict investments toward in-group versus out-group/stranger across 51 nations. The forest plot displays the effect sizes (Cohen’s *d*) of the difference in conflict investments between in-group members and out-group members/strangers across countries. Positive deviations from zero indicate stronger competition (i.e., more conflict investments) with an in-group member than an out-group member/stranger. Per country, out-group/stranger investments were calculated by averaging the investments toward out-groups + the average investments toward strangers. Dots represents effect size estimates and lines refer to the 95% confidence intervals (CI) of the estimates. Blue lines refer to defense (i.e., trying to avoid exploitation), and red lines refer to attack (i.e., trying to exploit the opponent). (**C**) Meta-analytic estimates of conflict investments toward in-group versus the rest across studies. The forest plot displays the effect sizes (Cohen’s *d*) of the difference in attack and defense between in-group members and all other opponents across all conducted studies.

Whereas in-group favoritism in cooperation is well-documented, we lack robust and uncontested evidence for the presence of in-group favoritism in competition (*6*, *17*, *32*, *34*). To address this gap, we focused on two aspects of conflict: when individuals try to outcompete others (viz., attack) and when they try to avoid being outcompeted (viz., defense) (*35*–*37*) (Fig. 1A; Materials and Methods). In these contest games, cooperation means that individuals do not invest anything, as any investment imposes a cost on their partner and reduces joint welfare (i.e., competition) (*37*, *38*). From the extant research on in-group favoritism (*2*, *7*, *15*, *39*), we initially hypothesized that people would be less competitive, or more likely to not invest anything, when interacting with an in-group member compared to an out-group member and stranger. Theoretically, if people are more inclined to create joint welfare when interacting with an in-group member, then it stands to reason that they also shy away from “wasting” personal and collective resources on competitions with an in-group member.

## RESULTS

### Humans as nasty neighbors

In study 1, participants made decisions in the attacker-defender contest (AD-C) (*35*, *37*). The contest models conflict and competition between an “attacker” and a “defender.” In the online experiment, participants were given an initial endowment of 10 “monetary units” (MU) and were assigned a role (attacker or defender; in the instructions, labeled as “person A” and “person B,” respectively). The value of each MU was standardized across societies and corresponded to 1 min of average wage in that country. Both attacker and defender had to decide how many of the 10 MU they wanted to invest into a so-called challenge pool or keep for themselves without knowing about the decision of their opponent. Whatever was invested in the challenge pool was lost. However, if the investment of the attacker was higher than the investment made by the defender, then the attacker “won” and earned any non-invested MU of the defender. In this case, the defender ended up with nothing. On the flipside, defenders successfully defended their remaining endowment if they invested at least the same or more into challenge pool. Hence, participants, depending on their role, could attempt to take away resources from the other person or defend against such attempts.

Participants from 51 different countries made 27 independent decisions to attack and 27 decisions to defend in a within-subject design (in a randomized order, see Fig. 1A) without feedback. In both roles, participants made one decision knowing that their opponent was a participant from the same country (in-group treatment), 25 decisions with opponents from the pool of nations included in the study (out-group treatment), and one decision with an opponent from an unknown country (stranger treatment). The out-group treatment was comprised of opponents of different nationalities to avoid the effect of nation-specific stereotypes (*7*, *12*). At the end of the study, participants were randomly assigned a role and an opponent and paid according to their decision.

Contrary to preregistered predictions, participants invested more resources in conflict with in-group members compared to out-group members and strangers (Fig. 1B), both when investing in attack (mixed-effects regression model, *b* = 0.346, *P* < 0.001) and when investing in defense (*b* = 0.283, *P* < 0.001). Results remain robust when considering out-group members and strangers separately, across various analytic strategies, and after controlling for gender, age, and education (tables S2 to S4 and figs. S3 to S5). The effect was in the same direction in all countries (Fig. 1B) and independent of country-level factors that were associated with in-group member favoritism in cooperation in earlier work, like the quality of institutions, pathogen stress, and economic wealth (fig. S5) (*40*). As a result, participants wasted more resources on conflict and earned less when interacting with an in-group member rather than an out-group member or stranger (attack: *b* = 0.467, *P* < 0.001; defense: *b* = 0.396, *P* < 0.001).

If competition is stronger for closer than distant others, then intensity of competition may also systematically decrease with geographical distance or cultural distance between opponents. To test this, we retrieved secondary data on geographical distance (*41*) and assigned a score that represented the bilateral distance between the biggest cities of the participant’s and their opponent’s countries (Materials and Methods). Extending the finding that individuals competed more with in-group than with out-group members and strangers, greater bilateral geographical distance was associated with lower conflict expenditure for both attack (*b* = −0.066, *P* < 0.001) and defense (*b* = −0.063, *P* < 0.001). Controlling for relevant individual and cross-societal differences, such as Gross Domestic Product (GDP), did not change results (table S5). While geographical distance might fail to account for similarities or differences across societies in terms of values, norms, or behaviors, we observed a similar pattern when replacing geographical distance with cultural distance in sociopsychological values between countries (*42*). In line with our experimental results, the more the individuals within a pair shared similar values, behaviors, or norms, the more the individuals from these two countries invested in attack (*b* = −0.039, *P* < 0.001) and defense (*b* = −0.023, *P* < 0.001) when interacting with each other (table S5).

Study 2 examined whether results from study 1 generalize beyond between-country online interactions and are also present in a lab-in-the-field experiment where individuals interacted within and between different ethnocultural groups residing within the same country (Kenya, *n* = 552; Materials and Methods). We focused on Kenya, as it has experienced marked costs of armed interethnic conflict. For example, since 2008, civil conflicts in Kenya have costed the life of more than 1500 people and triggered the displacement of up to 400,000 people (*43*). These interethnic conflicts often arise after political elections, where leaders historically represent members of different ethnocultural groups. Among those communities, the Luo and Kikuyu are the ones that have the greatest history of tensions and violent incidents (*44*). Our study included participants from these and (less opposed) ethno-cultural affiliations, allowing us not only to examine in-group favoritism in cooperation and competition but also to explore possible effects of conflict histories.

We used the same incentivized contest game as was used in study 1. Each participant made one decision with an in-group opponent that had the same ethnocultural affiliation as the participant, one decision with an out-group opponent that had a different ethnocultural affiliation than the participant, and one decision with a stranger whose ethnocultural affiliation was not given. We considered two communities marked by histories of conflict (Luo versus Kikuyu) and two communities with a more peaceful history (Kamba versus Luhya). We again find no support that shared group membership reduces conflict. Rather, as in study 1, people invested more in conflict with an in-group than out-group member and stranger (Fig. 1C), and this effect was independent of whether communities are characterized by current and previous histories of conflict. The effect was significant for defense (*b* = 0.178, *P* = 0.036), but not for attack (*b* = 0.046, *P* = 0.586; table S6), an asymmetry that replicates the findings for participants from Kenya in study 1 (see Fig. 1B).

Although the evidence against the “in-group favoritism in competition” hypothesis (as the theoretical flipside of in-group favoritism in cooperation) was unanticipated, research on animal behavior documented a similar tendency to compete more with close rather than distant conspecifics, a phenomenon dubbed the “nasty neighbor effect” (*45*–*50*). Accordingly, we preregistered a replication of our findings in a representative sample of UK participants in a follow-up study (study 3; *n* = 401, stratified by age, gender, ethnicity). Participants made decisions in attack and defense with opponents from different countries (per study 1; Materials and Methods). In line with the nasty neighbor effect, people invested more in both attack and defense when paired to an in-group member than an out-group member and stranger (Fig. 1C; attack: *b* = 0.285, *P* < 0.001; defense: *b* = 0.198, *P* < 0.001). As in study 1, participants were also less competitive toward members of geographically distant out-groups [*b* = −0.036, *P* = 0.01 for attack, and *b* = −0.022, *P* = 0.09 (nonsignificant) for defense; tables S7 and S8].

The AD-C in studies 1 to 3 has its equilibrium in a mixed strategy, meaning that what investment is in the individual’s best interest depends on what the other contestant does—investing in attack is, from the perspective of individual payoffs, wasteful or attractive when the defending party does invest much or nothing, respectively (*35*, *38*, *51*). These properties allow to more closely examine what individuals, paired with an in-group or out-group partner, were doing (for the mathematical underpinnings, see section S1.2). First, we see that the likelihood of not investing anything, akin to cooperating or not engaging in competition, is not different for in-group compared to out-group partners and strangers (fig. S2, A to D). This suggests that, in the decision to cooperate or not or to compete or not, people can be classified as neither in-group cooperators nor nasty neighbors (*52*). In line with this analysis, people were not nasty neighbors when asked to select a political in-group or out-group partner to compete with in a dot estimation task (for more detail, see section S5). Second, when we examined how much people invest relative to the mean expected investment assuming rational and risk-neutral agents that aim to maximize their expected payoffs, we see that individuals overinvest in both attack and defense more with an in-group partner than with an out-group partner and stranger (fig. S2, A and D). In sum, these additional analyses suggest that the nasty neighbor effect (i) is not about self-selecting oneself into competing (or cooperating) with in-group rather than out-group individuals but rather (ii) about the degree to which individuals compete within rather than between groups.

### The nasty neighbor effect coexists with in-group favoritism

Considering past research documenting in-group favoritism in cooperation, we further wanted to test whether and how higher nastiness toward in-group (versus out-group) members is related to favoritism toward in-group (versus out-group) members (*26*). In the dyadic contests, in-group favoritism in cooperation could have revealed itself in a greater likelihood of not investing anything in attack and defense when paired with an in-group member, something that we did not observe. However, as noted at the outset, in-group favoritism has often been observed when individuals can invest to the benefit of others, and the lack of evidence thus far is limited to the decision to cooperate or not (i.e., not invest or invest in attack or defense). Thus far, our studies did measure the degree of competition but not the degree of cooperation.

**Fig. 2.**
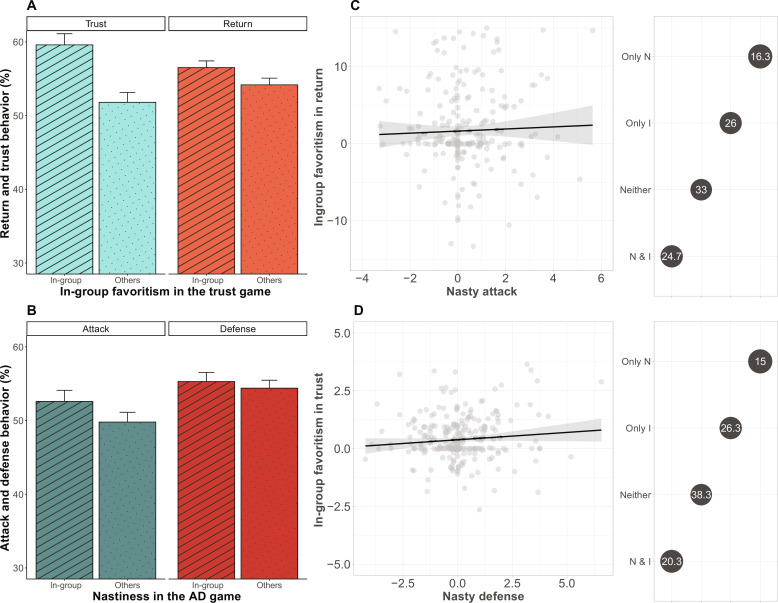
In-group favoritism coexists with neighbor nastiness. (**A**) In-group favoritism in the trust game. Bar chart showing mean differences (+ SE) in percentage between in-group partners and others (out-group and strangers) for transfers (viz., in-group favoritism in trust) and back-transfers (viz., in-group favoritism in trustworthiness). (**B**) Nastiness in the AD-C. Bar chart showing mean differences (+ SE) in percentage between investments with in-group opponents in attack and defense versus others (out-group and strangers). (**C**) In-group favoritism in trustworthiness is not associated with nastiness in attack. Scatterplot of the relation between conflict investments in attack toward in-group minus out-group members and strangers (nasty attack) and in-group minus out-group return of trust (in-group favoritism in trustworthiness). Bubble plot on the right shows the proportion of participants that can be classified as only in-group cooperator (“only I”), as only nasty (“only N”), as neither nasty nor in-group cooperator (“neither”), or as both nasty and in-group cooperator (“N & I”) based on their return and attack decisions. (**D**) In-group favoritism in trust is also not associated with nastiness in defense. Scatterplot of the relation between conflict investments in defense toward in-group minus out-group members and strangers (“nasty defense”) and trust behavior toward in-group minus out-group members and strangers (“in-group favoritism in trust”). Bubble plot on the right shows the proportion of participants that can be classified as only P, only N, as neither, or as N & P based on their trust and defense decisions.

In study 4 (*n* = 400), we examined whether in-group favoritism in cooperation and neighbor nastiness coexists within the same population across situations. Individuals interacted with someone from their own country (here United Kingdom) and 14 individuals from foreign countries (Material and Methods) (*15*). For each interaction, we assessed investment in the AD-C (Fig. 1A) and transfers and back-transfers in a standard trust game that measures forward-paying trust toward another person and trustworthiness (Materials and Methods and section S3.1). In the trust game, participants made decisions in two roles. As trustors, they were endowed with 5 MU, with each MU worth 1 min of average wage in the United Kingdom (0.20 pounds). Trustors were informed that they could send some or all of their MU to the trustee. The MU sent by the trustors were tripled, and trustees could then decide whether to return some of the MU received (see Materials and Methods). Both transfers (trust) and back-transfers (trustworthiness) were larger when paired to a fellow citizen than to a foreigner (Fig. 2A; trust: *b* = 0.327, *P* < 0.001; return: *b* = 2.330, *P* < 0.001; table S9), showing in-group favoritism in trust and trustworthiness. At the same time, we replicated the nasty neighbor effect in conflict investment: Individuals invested more in attack (*b* = 0.24, *P* = 0.003) and (nonsignificantly more) in defense (*b* = 0.071, *P* = 0.31) when paired to someone from their own rather than a foreign country (Fig. 2B, table S9, and fig. S2D).

Trust as operationalized with the trust game creates higher joint benefits for the actors, while reciprocity of trust leads to a fairer distribution of resources. Higher investments in attack and defense, on the other hand, reduce joint benefits and more likely increase inequality between parties. At the sample level, investment in competition (the aggregate of attack and defense investments in the contest game) was not correlated with investment in cooperation (the aggregate of transfers and back-transfers in the trust game) [correlation coefficient (*r*) = 0.023, *P* = 0.695]. More specifically, the (nasty neighbor) difference in in-group versus out-group attack did not predict in-group favoritism in trustworthiness (return to in-group–out-group partner) (Fig. 2C), and the (nasty neighbor) difference in in-group versus out-group defense was uncorrelated with in-group favoring, forward-paying trust (Fig. 2D). Instead, we observe quite some heterogeneity in our representative sample, with some individuals being in-group cooperators in the trust game and nasty neighbors in the contest game, some being in-group cooperators but not nasty neighbors, and some being neither in-group cooperators nor nasty neighbors (Fig. 2, C and D).

Findings thus far suggest that neighbor nastiness and in-group favoritism can coexist in situations where in-group favoritism is typically observed. To further substantiate this finding, we reanalyzed a cross-cultural dataset (*n* = 18,411; 42 societies) in which, on average, individuals were more cooperative when interacting with a national in-group member than with out-group member and stranger (*12*). In line with study 4, our reanalysis reveals substantial individual heterogeneity. While a majority of individuals were in-group cooperators (higher cooperation with in-group than with out-group and stranger: 48%, *n* = 8577), a large fraction of individuals were nasty neighbors (higher cooperation with out-group and stranger than with in-group: 32%, *n* = 5736), and this fraction was significantly higher than the fraction of people that did not discriminate between in-group and out-group members (20%, *n* = 3714; see section S3.2). Last, we also find that cross-cultural differences in in-group favoritism in cooperation do not correlate with cross-cultural differences in the nasty neighbor effect (*n*_countries_= 35, correlation between in-group favoritism and neighbor nastiness in attack: *r* = 0.02, *P* = 0.88; correlation between in-group favoritism and neighbor nastiness in defense: *r* = −0.19, *P* = 0.28). Together, these results underscore that in-group favoritism in cooperation does not imply in-group favoritism in competition and that, within and across individuals, in-group favoritism in cooperation does not predict how “nasty” one is toward an in-group member.

### Within-group status and resource scarcity bring forth nasty neighbors

In study 4, we show that people can be nasty neighbors and in-group–favoring cooperators at the same time, only nasty neighbors, only in-group–favoring cooperators, or neither. One possible explanation for this pattern is that within-group nastiness serves different and independent functions than in-group favoritism. Social interactions are often multifaceted and involve competition over cooperatively produced rewards. For example, some animals cooperate for hunting, but, in the process of the division of hunted prey, they often compete with each other (*25*, *53*). In addition, whereas in-group favoritism in humans correlates with prosocial preferences and group affiliation (*10*, *11*), the literature on the nasty neighbor effect in other animal species suggests that, among species-specific features, neighbor nastiness can help individuals to secure status ranking within their group (*45*, *48*) and (access to) a personal share of group resources (*45*, *47*, *54*). The behavioral data observed in study 1 are in line with this hypothesis. Individuals were not only more competitive toward in-group members than out-group members and strangers, but they were also more aggressive toward identifiable out-group members than unidentified strangers (table S2), a finding that is in line with this possibility.

**Fig. 3.**
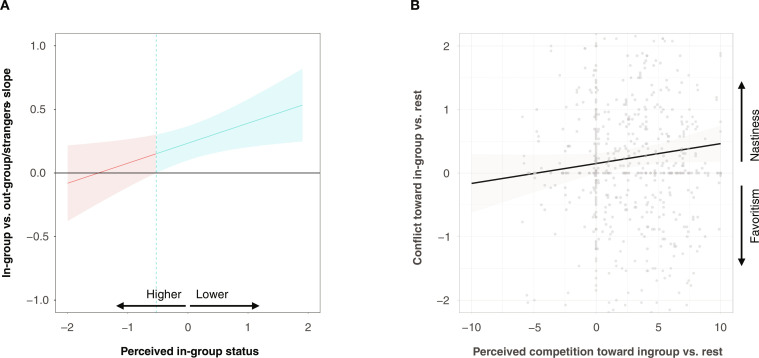
In-group status and perceived competition correlate with the nasty neighbor effect in humans. (**A**) In-group status concerns. Floodlight plot showing the regions of perceived in-group status (*x* axis, standardized) for which the effect of in-group versus others (*y* axis, positive values indicate support for the nasty neighbor effect) on conflict can turn significant. The vertical lines in the floodlight plot show the exact values at which significance begins and ends. Blue lines indicate significance at the 5% level. (**B**) Perceived resource scarcity. Scatterplot shows the association between perceived competition toward in-group members (minus out-group/strangers; rest) and the nasty neighbor effect (i.e., conflict investments with in-group individuals minus out-group members/strangers).

To shed further light on the mechanisms related to the nasty neighbor effect, we preregistered status concerns and resource scarcity as two plausible proximate reasons for the nasty neighbor effect in studies 3 and 4. Status concerns were measured with three items assessing how people perceive their status in their country. Perceived competition was elicited by asking participants to rate how much they thought their own well-being was influenced by competition over resources with each country participating in the study (more details in the Supplementary Materials). Consistent with the nonhuman animal literature, study 3 finds that the stronger neighbor nastiness, the more people perceived lower status within their nation (Fig. 3A; in-group versus out-group/stranger × perceived in-group status: *b* = 0.187, *P* = 0.015; table S10). Moreover, the nasty neighbor effect in study 3 was fully mediated by perceived resource scarcity, when controlling for beliefs and perceived similarity with the nation of the opponent (indirect effect: *b* = −0.232, *P* < 0.001; total effect: *b* = −0.254, *P* < 0.001; direct effect: *b* = −0.022, *P* = 0.740; table S11). Differences in how people perceived competition for scarce resources with in-group members compared to foreigners and strangers were positively associated with neighbor nastiness (*b* = 0.037, *P* = 0.027). Neither beliefs nor perceived similarities fully mediated or significantly interacted with the nasty neighbor effect (see section S4.1). We also found no evidence that the nasty neighbor effect interacted with individual-level characteristics that are often associated with in-group member favoritism [i.e., identification with one’s own group or nation, risk preferences, or prosocial preferences (*2*, *10*, *11*); Materials and Methods and table S10].

In study 4, we directly examined how in-group favoritism and the nasty neighbor effect were associated with social affiliation and identification on the one hand and perceived competition for scarce resources on the other. In line with study 3, perceived resource scarcity was positively associated with the nasty neighbor effect in attack and defense [for attack: *b* = 0.069, *P* = 0.035; for defense: *b* = 0.053, *P* = 0.073 (nonsignificant)] but not in-group favoritism in trust and trustworthiness (trust: *b* = −0.012, *P* = 0.527; trustworthiness: *b* = 0.032, *P* = 0.875). By contrast, national identification was associated with in-group favoritism in trust (*b* = 0.131, *P* < 0.001) and trustworthiness (*b* = 0.844, *P* = 0.01) but was not associated with the nasty neighbor effect in attack (*b* = −0.001, *P* = 0.971) and defense (*b* = 0.001, *P* = 0.999) (table S12). Together, these correlations support the possibility that in-group favoritism in cooperation and within-group nastiness coexist because each strategy relates to different elements of group living.

**Fig. 4.**
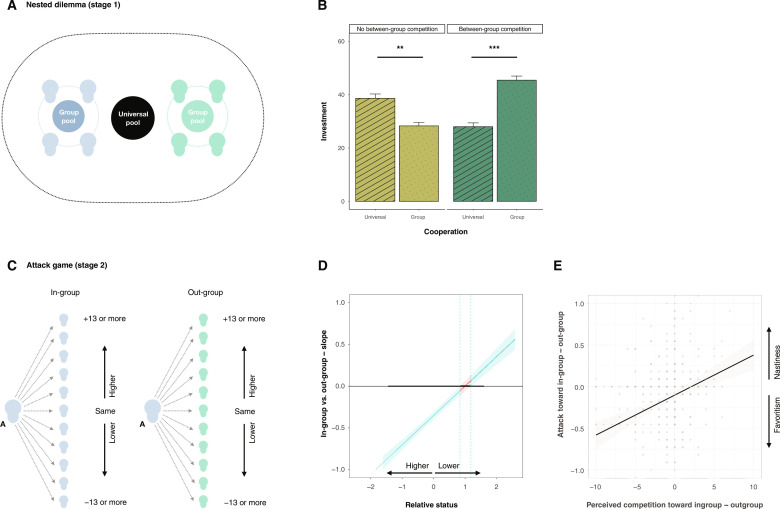
In-group cooperators and nasty neighbors in minimal groups. (**A** and **C**) Experimental setup of the nested social dilemma (top; stage 1, A) with an attack option (bottom; stage 2, C). (**B**) Between-group competition favors the emergence of in-group favoritism. Bar chart showing in-group favoritism and universal cooperation as mean percentage of the endowment contributed when competition is absent versus present. **P <* 0*.01, ***P < 0.001. (**D**) Status differences favor the emergence of within-group nastiness. Floodlight plot showing the regions of differences in status of the target of attack (*x* axis, standardized) for which the effect of in-group versus out-group (*y* axis) on attack becomes significant. The vertical lines in the floodlight plot show the exact values at which significance begins and ends. Blue lines indicate significance at 5% level. (**E**) Relative differences in perceived competition favor the emergence of a nasty neighbor effect. Scatterplot shows the association between perceived competition toward in-group members (minus out-group) and the nasty neighbor effect (i.e., attack of in-group members minus out-group members).

Our final study 5 (*n* = 552) had multiple goals including (i) an experimental manipulation of in-group status concern and competition to provide further causal evidence for the correlational findings of studies 3 and 4, (ii) a test of the nasty neighbor effect in an intergroup context where groups are strictly artificial and effects of stereotypes and conflict histories can be excluded, and (iii) an investigation on the boundary conditions that can give rise to in-group favoritism or within group nastiness. To that end, participants were assigned to one of two groups of four individuals each and were given an endowment for a two-stage nested social dilemma with a possibility to attack other individuals (i.e., reduce their earnings at a personal cost) (Fig. 4, A and B; Materials and Methods and section S4.3) (*9*, *17*). In stage 1, participants distributed 5 MU between a private pool that benefitted themselves only, an in-group pool that provided benefits to the in-group, and a universal pool that benefitted in- and out-group members alike. Each MU in the private pool was worth 1 MU to them and 0 MU to any other member in their own or in the other group. Each MU in the group pool would return 0.5 MU to each member in their own group, themselves included, and 0 MU to the members of the other group. Each MU in the universal pool (labeled “general pool” in the experiment) would return 0.5 MU to each member in their own group, themselves included, and 0.5 MU to each member in the other group. Accordingly, it was in the participant’s best interest to keep their 5 MU in the private pool (viz., free riding); contributions to the group pool were personally costly and benefitted the in-group (viz., in-group favoritism in cooperation), and contributions to the universal pool were (equally) personally costly and benefitted the in-group and out-group alike (viz., universal cooperation) (*9*, *17*). In addition, participants could earn a group bonus when the total contribution to their in-group pool exceeded that in the other group (between-group competition treatment). As predicted, we observed more in-group favoritism in cooperation in stage 1 when competition for a group bonus was present rather than absent (Fig. 4C; *t*_275_ = 5.77, *P* < 0.001).

In stage 2, participants could earn a personal bonus if they were the highest earning member in their own group (this treatment had no effects; table S13 and section S4.3). Participants could assign up to five “deduction points” to an in-group member and to an out-group member (*18*). Deductions reduced the target’s earnings at a 1-to-3 ratio (*55*). We operationalized member status in terms of their earnings from stage 1 relative to others in their in-group and in the out-group and elicited attack decisions in 11 possible scenarios. In five scenarios, the participant had lower earning status; in one, they had equal status; and in five, they had greater earning status (Fig. 4B and Materials and Methods). We find a significant earning status × target’s group membership interaction (*b* = 0.353, *P* < 0.001). Participants attacked lower-ranked in-group members more harshly than out-group individuals, akin to the nasty neighbor effect observed in studies 1 to 4. At the same time, participants attacked higher-ranking out-group individuals more harshly than higher-ranking in-group individuals, which can be interpreted as a form of in-group favoritism in competition toward higher (but not equal or lower) earning out-group individuals (fig. S6). These results resonate with earlier work on status contests in groups, finding that individuals are motivated to preserve their rank and are especially concerned about not being last (*56*, *57*). Hence, punishing lower status in-group members allows individuals to maintain their status within their group (even when this limits their possibility of winning an extra bonus). Results thus refine the conclusion from studies 3 and 4 that perceived lower status within group moderates the nasty neighbor effect. Furthermore, and also in line with studies 3 and 4, the nasty neighbor effect in attack was predicted by relative differences in perceived competition between in-group and out-group members (*b* = 0.048, *P* < 0.001; Fig. 4D).

## DISCUSSION

The tendency to trust and cooperate more with an in-group member than with an out-group member is both theoretically plausible (*19*, *39*) and empirically supported (*2*, *7*–*9*, *11*, *15*–*17*). As we showed here, however, in-group favoritism in cooperation does not mean that humans also compete more with distant rather than close others. Under the assumption of group-serving preferences, we should have seen higher trust and trustworthiness toward an in-group member as well as lower investments when these hurt or protect against an in-group member (compared to an out-group member). For the latter, we observed the opposite. Participants invested more in competing when paired to individuals sharing the same group affiliation than when paired to individuals sharing an “out-group” affiliation or being strangers with unknown affiliation. This nasty neighbor effect emerged for investment in AD-Cs, for a significant fraction of people in the prisoner’s dilemma game, and for costly attack of lower-ranking in-group members following public good provision [viz., antisocial punishment (*58*)]. In addition, while the nasty neighbor effect was robustly observed when group membership was based on nationality, with an observed effect size that is in line with that for in-group member favoritism in cooperation (*2*, *12*), we observed it too when individuals were identified by their ethnocultural affiliation or by arbitrary tags following random assignment to “minimal” groups.

Similar to research on cooperation (*12*, *15*), we find that cross-country differences in the nasty neighbor effect did not meaningfully covary with a set of relevant cross-cultural factors, such as rule of law or wealth (i.e., gross domestic product). Rather, we found meaningful variation when considering aggression and defense with in-group members. Specifically, in-group attack and defense correlated, at the country level, with hierarchical and egalitarian values and with wealth (see fig. S5). That said, future research is needed to further investigate the pervasiveness of the nasty neighbor effect across other contexts and further shed light on its cultural variation (*31*, *38*, *59*, *60*). For example, neither within-group nastiness nor in-group favoritism in competition was observed in tournaments where participants could self-select into competition with someone from their own political in-group or from a political out-group (section S5) [but see (*61*)]. Relatedly, while caution is needed when generalizing results to multifaceted day-to-day conflicts and competitions with neighbors and strangers, there are some notable parallels between the nasty neighbor effect observed here and observations outside the laboratory. First, past research found that individuals tend to anticipate threat from in-group members more than from out-group members, especially in collectivistic societies (*62*). Second, intragroup disputes and competitions in mobile forager societies can be equally or more violent than coalitionary aggression against other groups (*63*), and, at least, since the end of WWII, conflict and violence is as prevalent, if not more, within than between nation states (*34*). Last, as mentioned, the nasty neighbor effect documented here in humans has been observed in other species and across taxa, including social insects (*64*), group-living birds (*45*, *47*), and various mammals such as Eurasian beavers (*48*), banded mongoose (*46*), Diana monkeys (*50*), and black crested gibbons (*49*).

Whereas neighbor nastiness may emerge across a variety of settings, it may be limited to some types of groups and group affiliations. Groups have been defined in several ways, ranging from social relationships that connect two or more individuals, a set of individuals whose outcomes (or fitness benefits) are interdependent, to people that share formal or informal institutions (*19*, *65*). In individual decision-making as studied herein, groups have been conceptualized as cues (i.e., group tags or markers) that have been acquired through evolutionarily relevant and salient groups, and that individuals use to condition their choices (*39*). That said, the groups considered here (e.g., nationality and ethnocultural groups) are large, anonymous groups, mostly characterized by interactions among strangers and by weak bonds. At present, we need to exert caution when generalizing findings to group affiliations based on other group markers or groups characterized by face-to-face interactions such as small communities, friendships, or family ties [but see (*66*, *67*)].

Next to documenting neighbor nastiness in humans, we found that neighbor nastiness emerged independently from in-group favoritism in cooperation: The tendency to be more generous and trusting with individuals from one’s own group was unrelated to the tendency to compete more with individuals from one’s own group. Moreover, in several studies, we identified significant population heterogeneity in the co-occurrence of in-group favoritism in cooperation on the one hand and neighbor nastiness on the other; significant proportions of individuals could be classified as either in-group cooperators or nasty neighbors, while others were either in-group cooperators but not nasty neighbors or, conversely, not in-group cooperators but nasty neighbors. Such population heterogeneities and the observed coexistence of in-group favoritism in cooperation and neighbor nastiness are difficult to reconcile with prevailing theories on in-group favoritism and intergroup relations like social identity theory or bounded generalized reciprocity that, albeit for different reasons, both predict in-group favoritism in cooperation and, if anything, more rather than less competition with outsiders and distant others than with insiders and close others (*68*, *69*). Likewise, readers may wonder how current findings relate to prevailing theory on the evolution of cooperation in structured populations. For example, if we assume that groups frequently find themselves in recurrent intergroup competitions in which members of one group can benefit at the expense of members of other groups (*23*, *70*, *71*), then it should be beneficial for the individual to selectively cooperate with in-group members and cooperate less, or compete more, with out-group members. Intergroup competition and conflict would select against neighbor nastiness, as fiercer competition within than between groups would clearly disadvantage the in-group and reduce survival probabilities.

Whereas our findings are difficult to reconcile with prevailing theory in the social and behavioral sciences, a possible solution may be found in early work on inclusive fitness and population viscosity (*72*, *73*). The idea is that, in viscous populations, an individual is more related to a random neighbor than to a random individual in the population, and this stronger relatedness, or interdependency, may make individuals more frequently compete for status and shares of jointly created public goods within their own group than with distant strangers and individuals from out-groups. From this perspective, in-group cooperation can coexist next to neighbor nastiness because each serves different functions that may be differentially activated across time and space (*25*). If true, then ecological factors like resource scarcity can tip the balance from intragroup cooperation and mutual growth toward within-group competition and dissolution (*74*–*77*). Our experiment provided some initial support for this. In-group favoritism in cooperation was linked to group identification and affiliation, and neighbor nastiness was associated with status instabilities and resource scarcities. As such, we expect neighbor nastiness to emerge in games, group formations, and contexts that elicit status differences and competition over scarce resources and might be more pronounced in individuals who have less access to scarce resources or a lower status. Possibly, individuals optimize own benefits by flexibly switching whether to cooperate or compete, how much to cooperate or compete, and with whom to cooperate and with whom to compete.

In conclusion, current findings challenge the idea that in-group favoritism is a general and pervasive human tendency. In particular, in-group favoritism in competition may be confined to specific cases where groups experience zero-sum competition for scarce resources with other groups (*6*) or when histories of intergroup conflict and violence fuel spitefulness and revenge (*6*, *34*). In-group favoritism in cooperation makes groups wealthier and more likely to emerge victorious when competing with out-groups for scarce resources (*6*, *8*, *23*), yet, contrary to a widespread assumption, humans can also more fiercely compete with individuals who are close and part of their own group. While behaving as a nasty neighbor is costly and can undermine within-group solidarity, it can also secure the individual’s within-group status and privileged access to group resources. Rather than being either in-group cooperator or nasty, humans serve their groups or themselves by flexibly switching between favoring in-group members or acting as nasty neighbors.

## MATERIALS AND METHODS

### Study 1

The research and procedure were approved by the Psychology Research Ethics Committee of Leiden University (application number 2020-02-03-A. Romano-V1-2068).

#### Participants

We collected data from 12,863 participants across 51 countries (Algeria, Argentina, Australia, Austria, Belgium, Brazil, Bulgaria, Canada, Chile, China, Colombia, Czech Republic, Egypt, Finland, France, Germany, Greece, Hong Kong, Hungary, India, Indonesia, Ireland, Israel, Italy, Japan, Kenya, Korea, Malaysia, Mexico, Morocco, The Netherlands, Nigeria, Peru, Poland, Portugal, Romania, Russia, Saudi Arabia, Singapore, South Africa, Spain, Sweden, Switzerland, Taiwan, Thailand, Tunisia, Turkey, United Arab Emirates, United Kingdom, United States, and Vietnam). Participants were recruited through the Toluna Panel, including members of its third-party panel providers. Participants were stratified by age and gender. Our goal was to recruit 12,750 participants (~250 per society; section S2.1). A sensitivity power analysis showed that 250 people can detect a small effect size of *d* = 0.16 with 80% power (within-subject difference in conflict between in-group and out-group members/strangers).

#### Procedure and general design

The study was preregistered at https://osf.io/nf7ks/?view_only=1562f490520f4b5b90320185b2bbd445. The design consisted of two within-subject treatments related to the role of the participant (participant’s role: attacker versus defender; see below) and the opponent that the participant was interacting with (identified by the opponent’s nationality). The experiment was administered through an online survey. We wrote an English version of the survey and asked experts and professional translators to translate the survey. The procedure of the experiment was the same across all countries. After giving their informed consent, participants were asked to make 54 independent decisions, facing different opponents across the world. No feedback about others’ decisions was provided. Thereafter, participants also responded to several additional questionnaires, unrelated to this project, and asked to give information about their gender, age, and education. We note that throughout the instructions, we used neutral language and avoided terms like competition, defense, opponent, or conflict. Some of the data from study 1 have been used in another paper that examined different research questions and a different set of preregistered hypotheses (*78*). Specifically, the current paper tested preregistered hypotheses 2_a_, 2_b_, 3_a_, 3_b_, and 4_b_.

#### Treatments

For each decision, participants were assigned to interact with a person that was randomly selected from the pool of 51 nations included in the study. Before making their decision, they were informed about their partner’s nationality. We collected 54 decisions divided in two (randomized) blocks of 27 decisions, varying whether the decision was made in the role of attacker (attacker treatment) or in the role of defender (defender treatment). For each block, one decision involved interacting with a person of the same nationality, 25 decisions involved persons with a different nationality, and one decision involved an unidentified stranger (also, this order was randomized). Each nationality was randomly extracted once, such that participants could only make one decision as attacker and one decision as defender with a person of a specific nationality. The nationalities of the persons encountered in the second block matched those presented in the first block. Overall, the frequency of extracting persons’ nationalities was balanced across participants, such that we had an equal number of nation-nation pairs across the sample. We collected a total of 694,602 decisions among 12,863 participants from 51 countries.

#### Game-theoretic properties

Assuming rational selfish play, the AD-C has a unique Nash equilibrium in mixed strategies, such that players should randomize their investment (up to a certain threshold) to maximize their payoff. This means that there is not a single action that is clearly advantageous in the AD-C. The benefits of investing in conflict depend on the investments made by defenders and vice versa (*35*) (also see section S1.2).

#### Incentives

To make decisions comparable across nations in terms of earnings, each MU was worth 1 min of the average hourly wage in their country. Therefore, each participant started with an amount corresponding to 10 min of wage in their nation. Information of wage in each nation were retrieved from https://tradingeconomics.com/country-list/wages. Participants were paid for one role and one of their decisions in that role. We informed participants that they would make decisions in both roles and that, at the end of the experiment, we would randomly match each participant with another participant from the respective country and that their decisions would affect both their own earnings and the earnings of their randomly selected other party.

#### Geographical distance

Geographical bilateral distances measure city-level data to account for the geographic distribution of population inside each nation. Geographical distance is available for 225 countries and consists of the distance between two countries based on bilateral distances between the biggest cities of those two countries (*41*). We assigned a score to each decision interaction that represented the geographical distance between the participant and their opponent (section S2.1.3).

#### Analytic strategy

For the main treatment effect (attack versus defense), we used mixed-effects models in which participants (level 2) and nations (level 3) are two random factors. These models consider random intercepts for participants nested in nations. We analyzed data with R (lme4 package) (*79*). Individual differences variables (e.g., age and gender) were entered as level 2 controls.

### Study 2

The research and procedure were approved by the Psychology Research Ethics Committee of Leiden University (application number 2020-12-03-A. Romano-V2-2772) and by a local ethics committee, Strathmore University Institutional Ethics Review Committee (application number SU-IERC0958/20).

#### Participants

We collected data from 552 participants (*M*_age_ = 33.65, 27.71% women) in Nairobi, Kenya. Participants were recruited through the Busara Center for Behavioral Economics (https://busaracenter.org/). The design consisted of two within-subject treatments related to the role of the participant (participant’s role: attacker versus defender) and the opponent that the participant is interacting with (identified by the opponent’s ethnocultural affiliation). The experiment was conducted with a mobile lab with interactive tablets across diverse areas of Nairobi (Viwandani and Babadogo). We wrote an English version of the survey that was then translated to Swahili. As in study 1, instructions used neutral language, and we avoided terms like competition, defense, opponent, or conflict.

#### Procedure and general design

We recruited people from four ethnocultural communities: Kikuyu, Luo, Kamba, and Luhya. Participants gave their informed consent and were asked to make three independent decisions, facing different opponents (same, different, or unknown ethnocultural affiliation). No feedback about others’ decisions was provided. For members of the Kikuyu, the out-group member was always a Luo and vice versa. For members of the Kamba, the out-group member was always a Luhya and vice versa. Results are the same when controlling for type of pairing (table S6).

### Study 3

Study 3 was a preregistered replication (https://osf.io/j973y/?view_only=0d9bef6731364abbba068c199541f423) of study 1 performed in the United Kingdom. The research and procedure were approved by the Psychology Research Ethics Committee of Leiden University (application number 2022-09-26-A. Romano-V1-4261).

#### Participants, procedure, and general design

We collected a representative sample (based on age, gender, and ethnicity) of 401 participants from Prolific (*M*_age_ = 35.43, 51.62% women). Procedure and general design were the same as of study 1. To pay participants, we matched their decisions with the decisions made by the participants in study 1. The only difference with study 1 was the assessment of potential psychological mechanisms related to the nasty neighbor effect. After participants made their choices in the AD-C, they were asked to respond to questions related to generosity, national identity, risk preferences, perceived status, and perceived competition over scarce resources.

#### Generosity/altruism

We elicited generosity using two items of the global preference survey (*80*). In the first question, we asked participants how willing they were to give to good causes without expecting anything in return (0 = completely unwilling to do so to 10 = very willing to do so). The second question was a scenario in which participants were asked how much of a certain amount of money (700 GBP), unexpectedly received from a lottery, they would donate to a good cause.

#### Identity

We used the Single Item Social Identification (SISI) scale to measure national identity (*81*). Participants were asked how much they identify with their nationality on a seven-point Likert scale.

#### Perceived similarity

We administered a scale of perceived similarity. We used the definition of sociopsychological distance and asked participants about their perception of similarity of out-group countries (“For each country, please indicate to what extent you perceive this country similar in terms of values, beliefs, and behaviors; 0 = very dissimilar to 10 = very similar”).

#### Risk preferences

We elicited risk preferences using one qualitative item from the global preference survey (*80*). The qualitative item asks for the respondents’ self-assessment of their willingness to take risks on an 11-point scale (0 = completely unwilling to take risks to 10 = very willing to take risks).

#### Perceived status

We administered a measure of social insecurity with both in-group and out-group members (*82*). The scale is composed of three items (Cronbach’s alpha = 0.78), and participants were asked how much they agree with each statement on a 5 points Likert scale (1 = strongly disagree, 5 = strongly agree; example: “I feel that my status in my country (among foreigners) is threatened”).

#### Perceived competition over scarce resources

We administered a scale of perceived competition by asking participants to rate how much they think their own well-being is influenced by competition with that country (0 = not at all, 10 = very much).

### Study 4

Study 4 was designed to assess whether parochialism and the nasty neighbor effect could be observed in the same set of individuals. The general research design and procedure were approved by the ethics committee of the Faculty of Arts and Social Sciences at the University of Zurich (22.10.5) and preregistered at https://osf.io/qt7zy/?view_only=9f0ae5877024489daca434b08aba7c3c.

#### Participants, design, and procedure

We recruited 300 participants through Prolific, stratified by age, gender, and ethnicity. Participants made decisions in a 2 (situation: competitive versus cooperative) × 15 (partner’s nationalities) × 2 (role in competition: attacker versus defender) × 2 (role in cooperation: trustor versus trustee) within-subject design. A sensitivity power analysis showed that 250 people with 80% power can detect a small effect size of *d* = 0.16 (within-subject difference). Participants provided informed consent and read instructions of the two-player AD-C or of the trust game (the order of the two games was randomized). In the AD-C, after responding to comprehension and attention questions, participants made 16 independent decisions as attackers and 16 independent decisions as defenders in randomized order (methods were identical to those in study 3). In the trust game, after responding to comprehension and attention questions, participants made 16 independent decisions as trustors and 16 independent decisions as trustees in randomized order. Roles were labeled with a neutral term (i.e., “invest person” and “return person”). As trustors, they were endowed with 5 MU, with each MU worth 1 min of average wage in the United Kingdom (0.20 GBP). Trustors were informed that they could send some or all of their MU to the trustee. The MU sent by the trustors were multiplied by 3. The trustees could then decide whether to return some of the MU received. As interactions were not simultaneous, trustees were asked to state how much they would return for each potential investment made by trustors.

In each game and for each role, participants made 16 decisions. Each decision was made with a partner of a different country (including the own country). The specific nationality of the partner were selected from the pool of participating countries in the previous 15-society studies: Argentina, Brazil, China, Germany, Indonesia, Italy, Japan, Korea, Poland, Russian Federation, Spain, Taiwan, Turkey, United Kingdom, and United States (*15*). All participants also interacted once with a person of the own nation and an unidentified stranger. We incentivized decision-making in both games by selecting, at the end of the data collection and for each participant, one game and matched pairs of participants by randomly selecting one decision and paying them based on the outcome of that decision.

#### Measures

Following decision-making, we collected measures of demographics, perceived in-group status, perceived competition of scarce resources, and national identification, using the same measures as used in study 3.

### Study 5

The research design and hypotheses were preregistered (https://osf.io/qrw5x/?view_only=447b15e646e74623acd2af3e04066060) and received ethics approval from the ethics committee of the Faculty of Arts and Social Sciences at the University of Zurich (22.10.5). The experiment involved no deception and was fully incentivized.

#### Research design and experimental procedures

We examined contribution decisions in a two-stage nested social dilemma with an attack option (*9*, *17*), with participants being randomly assigned to the conditions of a 2 (between-group competition: absent/present) × 2 (within-group competition: absent/present) between-subjects factorial. We collected data from a representative sample (based on age, gender, and ethnicity) of 552 participants from Prolific (*M*_age_ = 35.43, 51.62% women). Participants were organized in two groups of four each (henceforth “your group” and “the other group”). Participants were informed that they would receive MU for a two-stage decision-making task, that decisions would affect personal earnings alongside those of others in their own group and in the other group, and that their identity would never be revealed to other participants. The rules and procedures of stage 1 and stage 2 were thoroughly explained, and comprehension was verified before participants could proceed with decision-making. Following decision-making, participants responded to a short survey, were debriefed, and were paid.

#### Nested social dilemma (stage 1) with an attack option (stage 2)

For stage 1, participants distributed 5 MU (1 MU = 0.1 GBP) across a private pool, their group pool, and a universal pool. For stage 2, participants received another 20 MU and could use up to 5 MU to deduct earnings from someone else in their own group and in the other group. Each MU assigned to someone else would cost the participant 1 MU and reduce the target’s earnings by 3 MU (*55*). Earnings from stage 2 would thus be the total of 20 MU received, minus the deduction points assigned, minus the deduction points received × 3.

#### Between-group competition (stage 1)

Before decision-making, we explained that participants could earn a group bonus for stage 1 and a personal bonus for stage 2. The group bonus introduced the presence (versus absence) of competition between one’s own group and the other group. Specifically, participants in the “intergroup competition absent” treatment were informed that the group bonus of 4 MU would be decided on the basis of a lottery with 50% chance of getting the bonus (giving each group member one additional MU). In contrast, participants in the “intergroup competition present” treatment were informed that the stage 1 group bonus would be earned by the group who had made the largest investment in their respective group pool (or a coin flip in case of a tie). If their group put more in the group pool than the other group, then each member in their group would thus earn one additional MU.

#### Within-group competition (stage 2)

Participants in the “within-group competition absent” treatment were informed that, after stage 2, one member in each group would be randomly selected to earn a personal bonus of 4 MU. In contrast, participants in the “within-group competition present” treatment were informed that, after stage 2, the individual in each group who earned the most from stage 1 and stage 2 combined would earn a personal bonus of 4 MU.

#### Measuring cooperation in stage 1

Following instructions, participants received a short summary of the stage 1 and stage 2 decision-making, alongside the (treatment-dependent) rules for earning the group and personal bonuses. They then distributed 5 MU across their private, group and universal pool, and estimated how much the other three members of their group combined invested into their group pool (range, 0 to 15) and into the universal pool (range, 0 to 15). Beliefs were incentivized with 0.25 GBP for each correct estimate.

#### Measuring attack in stage 2 conditional on earning status

Next, participants assigned up to 5 MU as deduction points to an individual in their own group and to an individual in the other group (order counterbalanced). For each target individual, we asked participants to assign deduction points between 0 and 5 MU given that this other person, in the first stage, earned (i) “13 or more than you,” (ii) “10 to 12 more than you,” (iii) “7 to 9 more than you,” (iv) “4 to 6 more than you,” (v) “1 to 3 more than you,” (vi) “the SAME as you,” (vii) “1 to 3 less than you,” (viii) “4 to 6 less than you,” (ix) “7 to 9 less than you,” (x) “10 to 12 less than you,” and (xi) “more than 13 less than you.” We explained that we would match participants and implement the deduction decision to the actual earning configuration that applied. At the end of this task, participants estimated how much deductions they would receive from in-group and out-group members. They were paid 0.25 GBP if they were correct.

#### Measuring perceived competition

Perceived competition was measured as in studies 3 and 4.

## Supplementary Materials

### This PDF file includes:

Sections S1 to S5 Figs. S1 to S6 Tables S1 to S13 Legend for data S1 References
