# Supplementary material for: The nasty neighbor effect in humans
Source: Sci Adv. 2024 Jun 26;10(26):eadm7968. doi: 10.1126/sciadv.adm7968 (PMC11204206; doi:10.1126/sciadv.adm7968)
Supplement: Supplementary file 1 — Sections S1 to S5 Figs. S1 to S6 Tables S1 to S13 Legend for data S1 References [file sciadv.adm7968_sm.pdf]

Science Advances
AAAS

Supplementary Materials for

The nasty neighbor effect in humans

Angelo Romano *et al.*

Corresponding author: Angelo Romano, a.romano@fsw.leidenuniv.nl

Sci. Adv. **10**, eadm7968 (2024)
DOI: 10.1126/sciadv.adm7968

The PDF file includes:

Sections S1 to S5
Figs. S1 to S6
Tables S1 to S13
Legend for data S1
References

Other Supplementary Material for this manuscript includes the following:

Data S1

1. Behavioural games for ingroup favouritism in cooperation and competition

1.1. Introduction

A variety of experimental games have been employed to identify ingroup favouritism (*2*, *8*). In its simplest form, a game involves two agents A and B each with a binary choice that is taken simultaneously and after which the game ends. Individual payoffs are a function of the combination of both agents' choices. As an example, consider the Prisoner's Dilemma (PD). In the two-player PD, each player must choose between an action often labelled as cooperation (C) and another action labelled defection (D). Each player benefits personally most from choosing D rather than C, and the unique pure Nash Equilibrium is reached when both players choose D. In equilibrium, however, each player earns less compared to when both had chosen C. In the PD, the C-C combination maximizes joint payout and is 'pareto efficient' (*51*), but is not an equilibrium of this game. Choosing D in the Prisoner's Dilemma is a dominant strategy. Ingroup favouritism is revealed as a stronger propensity to choose C than D when interacting with an ingroup rather than an outgroup member or stranger.

1.2 Attacker Defender Contest Game

The attacker defender contest (AD-C) is special case of a class of Tullock contest games that model competition, or 'rent-seeking' between individual and groups and has strong similarities to related games like tug-of-war and pre-emptive strike games (*35*, *38*, *51*, *83*). The contest has two players A (for attacker) and D (for defender) each with an endowment e from which they can invest x in a contest. As is typical for contest games, any investment is wasted. Similar to the Prisoner's dilemma game, there is a pareto efficient state where both agents do not invest anything (Attacker x_A = Defender $x_D = 0$). However, when investments in attack exceed those in defence ($x_A > x_D$), the attacker earns the non-invested resources from the Defender ($e - x_D$) leading to a payoff of 2e - x_A - x_D; in this case the defender earns 0. In all other cases, where $x_A \leq x_D$, attacker and defender earn their non-invested resources (i.e., $e - x_A$ and $e - x_D$, respectively). For rational selfish players, the peaceful solution ($x_A = x_D = 0$) does not constitute a Nash equilibrium. When $x_D = 0$, an Attacker should simply invest one unit ($x_A = 1$) to win the conflict (in other words, $x_A = 0$ is strictly dominated by $x_A = 1$ in this case). Yet, if $x_A = 1$, the Defender should respond with $x_D = 1$, which then changes the optimal response of the Attacker to $x_A = 2$. Note that because of these properties, the AD-C is an asymmetric game in that a player's incentives for a particular strategy change when they change roles (this is not the case in symmetric games like, e.g., the PD).

Figure S1. Equilibrium strategies in the attacker-defender contest for equal endowments (*e* = 10). Adopted from Meder et al. (*84*).

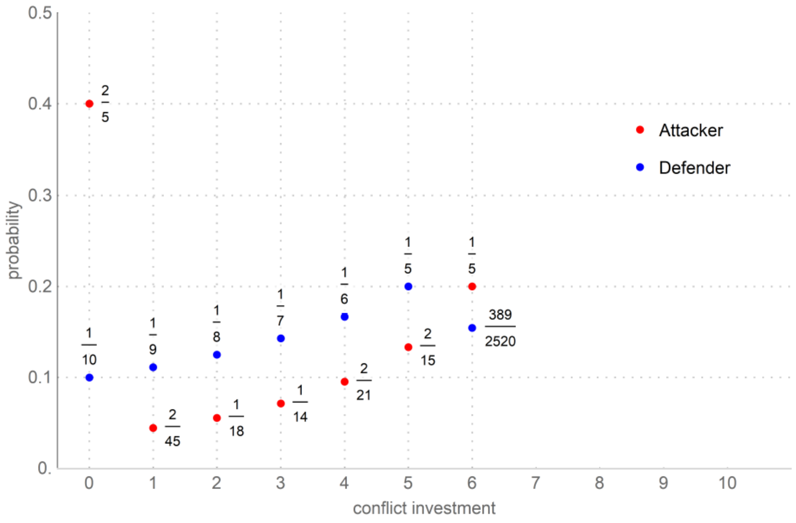

A formal analysis of the equilibrium properties of the AD-C reveal its Nash equilibrium in mixed strategies ((*35*, *84*); Figure S1). Compared to the PD, there is no pure Nash equilibrium. With an equal starting endowment of *e* = 10 for attackers and defenders, we denote p(X) as the probability of investing X by attackers, and p(Y) the probability of investing Y by defenders. A strategy assigns a probability value for each possible action (i.e., investment). In equilibrium attackers should choose:

$p(X = 1) = 2/45$,
$p(X) = p(X - 1)*[(12 - X)/(10 - X)]$ for $2 \leq X \leq 6$,
$p(X = 0) = 1 - [p(X = 1) + \ldots p(X = 6)] = 0.4$,
$p(X) = 0$ for $X \geq 7$

i.e.; $P(0) = 0.4$, $P(1) = 0.0\overline{4}$, $P(2) = 0.0\overline{5}$, $P(3) \approx 0.0714$, $P(4) \approx 0.0952$, $P(5) = 0.1\overline{3}$, $P(6) = 0.20$, $P(7) = 0$, $P(8) = 0$, $P(9) = 0$, $P(10) = 0$.

Defenders should choose:

$p(Y) = 1/(10\text{-}Y)$ for $0 \leq Y \leq 5$,
$p(Y = 6) = 1 – [p(Y = 0) + \ldots + p(Y = 5)] = 0.15$,
$p(Y) = 0$ for $Y \geq 7$
i.e.; $P(0) = 0.1$, $P(1) = 0.1\overline{1}$, $P(2) = 0.125$, $P(3) = 0.1428$, $P(4) = 0.1\overline{6}$, $P(5) = 0.2$, $P(6) = 0.15$, $P(7) = 0$, $P(8) = 0$, $P(9) = 0$, $P(10) = 0$.

This means that, contrary to the PD, in the AD-C there is not a clearly advantageous action. The personal earnings from investing in conflict depend on the investments made by the opponent, and vice versa. The best-response of defenders is to match attackers’ investments, whereas for attackers, the best response

would be to invest exactly either one unit more than the defenders or to not invest in attack at all, depending on whether the remaining capital not invested by attackers and defenders is large enough to make an attack investment worthwhile. Ingroup favouritism in the AD-C would mean stronger conflict investment when interacting with an outgroup individual or stranger, than with an ingroup member. In sum, unlike the prisoner's dilemma, the attacker-defender game does not have a pure Nash equilibrium, and it is characterized asymmetric payoff functions for attacker and defender.

The equilibrium properties of the attacker-defender game allow to identify that rational, risk-neutral, expected payoff maximizing agents on average invest i_{att} = 2.62 in attack, and i_{def} = 3.38 in defence. Furthermore, we can identify that for rational payoff maximizing agents, $i > 6$ (with e = 10, as in the current studies) are out-of-equilibrium and should never be played. With these game-theoretic 'benchmarks', we can conclude that in Study 1, people 'over-invested.' The mean investment in attack with in-group (out-group; strangers) is 5.70 (5.34; 5.09 MU), and the mean investment in defence with in-group (out-group; strange) is 5.71 (5.42; 5.27).

The mixed-strategy equilibrium of the attacker-defender contest implies that mean investments, across trials and with different opponents, emerge because of some mixing of investments (for the game-theoretic probabilities with which investments are played, see Figure S1 here above). In our studies 1, 3 and 4 (where, in contrast to Study 2, participants made a number of investment decisions and distributions can be meaningfully assessed), we indeed see that the average 'over-investment' in attack and defence emerges because out-of-equilibrium investments are played more than expected under the assumptions of rational payoff maximization (Figure S2.a to S2.c).

Recall that not investing at all in conflict, $i_{att} = i_{def} = 0$ is preserving social welfare and can be considered the maximally cooperative action to choose. Rational agents are expected to choose $i = 0$ sometimes and also in our studies, we observe $i = 0$ actions. However, both i_{att} and i_{def} are above 0 more often than would have been expected under the assumptions of rationality. Moreover, and crucially, we observe $i = 0$ in equal proportions when individuals are paired with an opponent from their in-group, an out-group or with a stranger. This suggests that the nasty neighbour effect is not so much about whether someone competes (or cooperates), but rather about how much one invests in competing. We return to this when reporting the complementary 'tournament' study in section 5 here below.

Figure S2. Proportions of investments made for each possible investment level (0, 1, 2, … 10) when agents play in equilibrium (open bars), and when participants are paired with an ingroup member (solid bars), or an out-group member or stranger (dashed bars). (a) Study 1 for attack (left) and defence (right) with out-group and stranger combined; (b) Study 1 with out-group and strangers separated for attack (left) and defence (right); (c) Study 3 for attack (left) and defence (right); (d) Study 4 for attack (left) and defence (right).

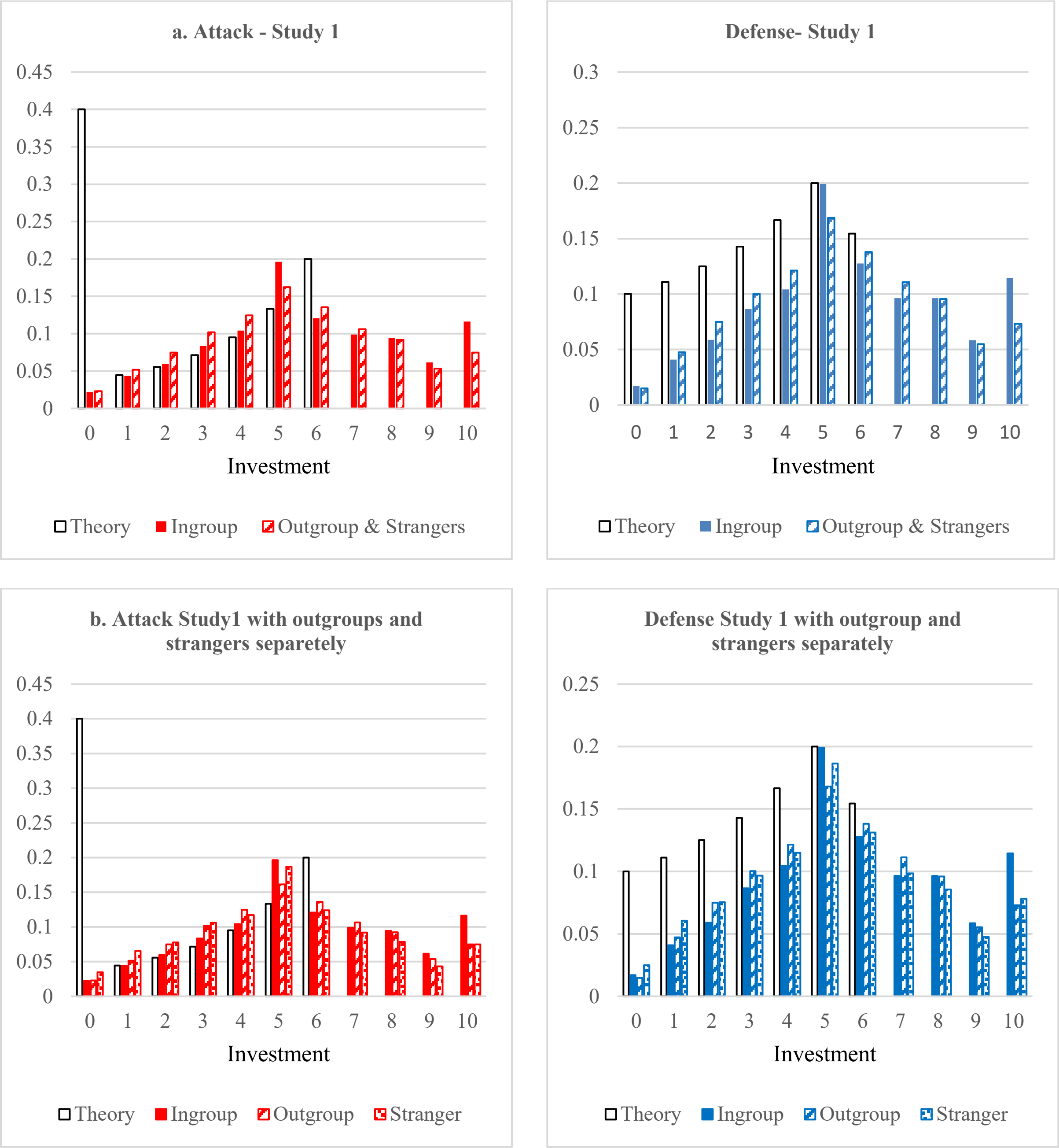

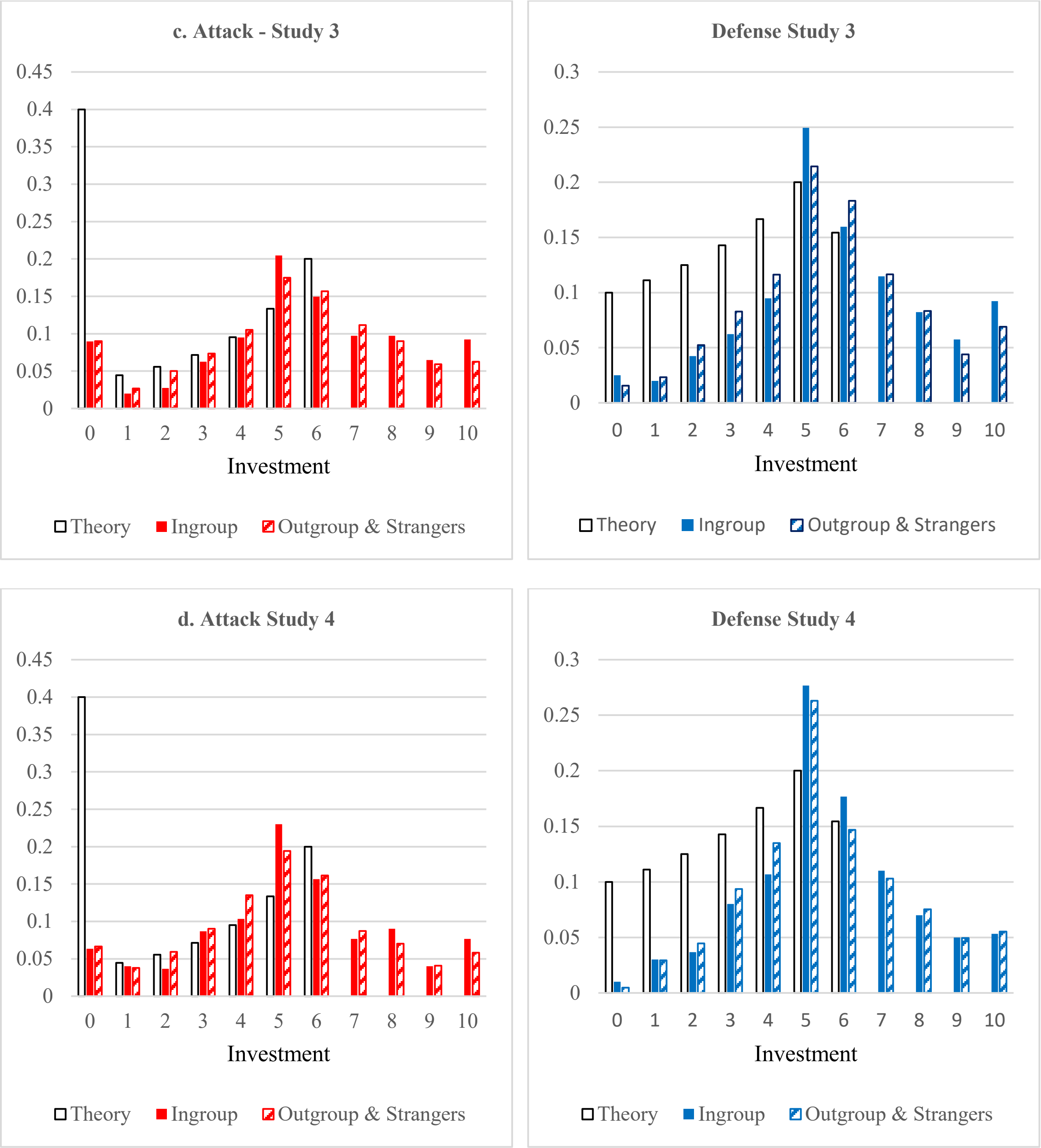

Note. Theory = predicted frequency proportion for each investment level according to the mixed-strategy equilibrium. Ingroup = observed frequency proportion for each investment level in interactions with ingroup members. Outgroup = observed frequency proportion for each investment level in interactions with outgroup members. Strangers = observed frequency proportion for each investment level in interactions with strangers.

1.3 Trust Game

In its standard form (*85*), the TG has two players, the investor (I) and the trustee (T), and involves two stages. In Stage 1, the investor decides how much out of endowment e to transfer to the trustee. Transfer x (with $0 \leq x \leq e$) is tripled and, in Stage 2, the trustee decides how much to transfer back (y) to the investor (with $y \leq 3x$). This ends the game, with the investor earning $e - x + y$, and the trustee earning $3x$ - y. Strict payoff maximizing, rational players in the TG should neither transfer ($x = 0$) nor back-transfer ($y = 0$).

Transfers are assumed to reflect how much the investor trusts the trustee. Back-transfers are assumed to reflect the trustee's generosity or concerns for reciprocity. Ingroup favouritism in the TG would manifest in larger transfers and/or back-transfers when interacting with an ingroup partner, than with an outgroup individual or stranger (*2*). Stronger ingroup favouritism in transfers reflect investors trusting and/or being more generous towards ingroup partners more than outgroup partners or strangers. Stronger ingroup favouritism in back-transfers reflect trustees are more generous towards ingroup partners compared to outgroup partners and strangers (*15*).

1.4. Nested-Social Dilemma

Like the classic prisoner's dilemma, the attacker-defender contest and trust game are so-called one-level games between two individuals. Ingroup favouritism has been examined also in two-level games, where individuals are nested in groups and groups are nested in an overarching collective (for a review, see (*6*)). One example is the nested social dilemma (*9*, *17*) (NSD). Individuals (e.g., 8) are organized in two groups of (usually) equal size, an ingroup and an outgroup, and are given an endowment e which they distribute at their own discretion across three pools – private, club, and universal. Allocations to private benefit the individual only and no one else (in our Study 5, one unit in private was simply worth 1 unit for payout). Allocations to club benefit all ingroup members, the individual included, and no one else. In our Study 5, one unit in club returned 0.5 to each ingroup member, and 0 to outgroup members. Allocations to universal benefit all individuals in the in- and outgroup alike. In our Study 5, one unit in collective returned 0.5 to each individual in the in- and the outgroup. Contributions to club are sometimes referred to as parochial, or ingroup cooperation, and to universal as universal cooperation.

Because allocations to both club and universal are (equally) costly to the individual (i.e., their marginal return is lower than allocations to private), strict payoff maximizing agents in the NSD should allocate their entire e to private, and nothing to club or collective. Like the PD, the NSD thus has a single pure Nash equilibrium in choosing D (i.e., allocating all units to private). And like the PD, it is pareto efficient and collectively most beneficial to allocate the entire e to collective. Under the current parameters, allocating to club is intermediate between strict payoff maximization and pareto efficiency, and reflects ingroup favouritism. In the NSD, ingroup favouritism, among other things, maximizes ingroup welfare, increases

the probability that the ingroup emerges as wealthier than the outgroup, and prohibits that the ingroup is being 'exploited' by the outgroup.

1.5 Costly Punishment

Punishment, conditional on observed actions or outcomes, has been widely investigated in the social dilemma literature (e.g., (*55*)). In economic games, punishment is often operationalized as a costly action that a player can take to reduce the earnings of another player after observing their choice ('peer punishment'). If punishment is costly for the punishing party, rational-selfish players should not dedicate any resources to punishment in one-shot games since punishment reduces own earnings. Nevertheless, peer punishment is often observed in the laboratory (*55*). In the NSD, ingroup favouritism in punishment (or attack) can be revealed by looking at the willingness to punish conditional on the outcome or earnings of the punished. If punishment choices differ for the same outcome depending on the group membership of the punished, it shows that punishment is not impartially used but influenced by what group the person belongs to ((*18*), 'parochial punishment'). Importantly, punishment can be employed to decrease or increase the gap between own earnings (after Stage 1) and others' earnings. Higher punishment towards ingroup or outgroup members can thus change the relative standing (in terms of earnings) within or between groups.

2. Humans as nasty neighbours

2.1 Study 1

The study comprised a total of 51 nations, and a sample size of 12,863 subjects. There was no specific screening for the selection for countries, other than trying to diversify the variability in terms of the economic, institutional, and cultural context as much as possible. We selected all countries that were available in the Toluna panel. The full list is presented on the following page.

Table S1. List of countries included in Study 1

Country	*N*	*% Women*	M_{age} *(SD)*	*Language*
Algeria	198	29.29	36.77 (10.76)	Arabic
Argentina	231	55.41	37.21 (12.52)	Spanish
Australia	254	53.94	44.18 (12.71)	English
Austria	254	52.36	39.39 (13.38)	German
Belgium	232	52.16	41.25 (13.16)	French & Dutch
Brazil	253	50.99	37.72 (12.26)	Portuguese
Bulgaria	251	43.03	38.73 (11.96)	Bulgarian
Canada	277	53.79	44.68 (12.72)	French & English
Chile	234	62.82	34.49 (12.06)	Spanish
China	244	47.54	37.77 (11.34)	Simplified Chinese
Colombia	231	50.22	38.73 (12.77)	Spanish
Czechia	257	50.97	39.41 (13.65)	Czech
Egypt	247	41.30	35.08 (11.03)	Arabic
Finland	276	51.81	41.69 (12.72)	Finnish
France	234	52.56	43.66 (11.88)	French
Germany	232	51.95	45.54 (12.35)	German
Greece	229	54.15	38.42 (12.17)	Greek
Hong Kong	252	50.00	38.69 (12.47)	English & Chinese
Hungary	260	51.92	40.84 (14.29)	Hungarian
India	225	50.22	37.65 (12.41)	English
Indonesia	231	48.05	37.90 (11.89)	Indonesian
Ireland	266	57.14	40.67 (11.76)	English
Israel	254	47.03	40.07 (13.11)	Hebrew
Italy	258	50.78	40.91 (12.98)	Italian
Japan	226	39.38	42.98 (11.18)	Japanese
Kenya	249	58.23	31.38 (9.32)	English
Korea	260	45.00	40.59 (11.85)	Korean
Malaysia	258	45.35	37.28 (11.98)	English & Malay
Mexico	250	50.80	37.57 (12.01)	Spanish
Morocco	253	35.17	30.77 (9.39)	Arabic
Netherland	239	53.56	42.5 (12.51)	Dutch
Nigeria	226	71.24	30.3 (10.63)	English
Peru	267	52.43	34.09 (11.11)	Spanish
Poland	252	48.81	38.45 (13.05)	Polish
Portugal	271	51.29	40.25 (13.16)	Portuguese
Romania	258	49.22	39.41 (13.51)	Romanian
Russia	237	51.05	40.77 (12.43)	Russian
S. Arabia	236	47.88	34.03 (9.76)	Arabic & English
Singapore	265	47.55	39.78 (12.53)	English
S. Africa	253	52.57	37.48 (12.86)	English
Spain	254	46.46	40.45 (12.22)	Spanish
Sweden	241	50.21	43.59 (13.26)	Swedish
Switzerland	283	53.71	41.59 (12.97)	German & French
Taiwan	290	46.55	36.88 (12.06)	Chinese
Thailand	311	50.16	39.84 (13.02)	Thai
Tunisia	299	39.46	40.51 (11.91)	French & Arabic
Turkey	270	52.96	35.33 (11.41)	Turkish
UAE	270	46.67	34.24 (10.28)	Arabic & English
UK	262	53.82	43.02 (13.29)	English
USA	229	51.53	44.00 (14.04)	English
Vietnam	274	51.46	33.42 (9.68)	Vietnamese
**Total**	**12.863**	**50.04**	**38.86(12.29)**	

2.1.1. Treatment effects. First, we present the models with the main treatments: ingroup (vs outgroup & stranger) for both attack and defence. We excluded participants that did not responded correctly to the comprehension checks in their first attempt and that failed an attention check in which they were asked not to respond to a Likert-style question (~23%). The ingroup (vs outgroup & stranger) variable is a dummy variable with decisions with outgroup members and undefined strangers coded as 0, and decisions with ingroup members coded as 1. In these models, participants and countries were random intercepts. Contrary to our pre-registered hypotheses, we find that people invested more in attack and defence with opponents from the same country, compared to opponents from foreign (i.e., outgroup) countries and strangers (Table S2). Results remain the same even when we separately consider decisions with outgroup members (attack: $b = -0.343$ $p < .001$; defence: $b = -0.283$, $p < .001$) and decisions with strangers, only (attack: $b = -0.606$, $p < .001$; defence: $b = -0.447$, $p < .001$). Including excluded participants in the analyses do not meaningfully affect the results.

In Table S2 we also show whether investments in attack or defence towards outgroup members differ from investments toward strangers. In the cooperation literature, such contrast is often used to disentangle whether parochial cooperation is driven by ingroup favouritism or outgroup derogation (*15*). However, given that we find that parochial competition does not occur in the first place, this contrast can no longer be interpreted in relation to the difference in competition between ingroup and others. Nonetheless, we find that people invest more in both attack and defence with opponents from outgroup countries compared to unidentified strangers.

Table S2 Mixed-effect model of treatment effects on investment decisions. Tests are two-sided. $N_{\text{observations}} = 363{,}231$; $N_{\text{subjects}} = 12{,}863$, $N_{\text{countries}} = 51$. Ingroup = 0, Outgroup & stranger = 1.

	Investment in Attack			Investment in Defence		
Contrast	*b*	*SE*	*p*	*b*	*SE*	*p*
Ingroup vs outgroup & stranger	0.346	0.015	<0.001	0.283	0.014	<0.001
Outgroup vs stranger	-0.269	0.014	<0.001	-0.171	0.014	<0.001

One potential concern related to the results of the main regression model presented in the manuscript and in Table S2 is that people made several decisions with different outgroup members, and this might have affected our results. We run two different models to test the robustness of the nasty neighbour effect, and to shed more light on its pervasiveness. In one model, we added a *random intercept for country of the opponent* that considers the dependencies in the choices made with opponents of outgroup countries. This is a very conservative approach as our main hypothesis pertains to the effect of ingroup vs outgroup members, independently of stereotypes. Nonetheless, we find that even with such conservative

specification, people still significantly invest more in attack and defence with ingroup members, than outgroup members and strangers (Table S3).

Table S3 Mixed-effect model of treatment effects on investment decisions. Tests are two-sided. $N_{observations}$ = 363,231; $N_{subjects}$ = 12,863, $N_{countries}$= 51, $N_{opponents}$= 53. Ingroup = 0, outgroup & stranger = 1.

	Investment in Attack			Investment in Defence		
Contrast	*b*	*SE*	*p*	*b*	*SE*	*p*
Ingroup vs outgroup & stranger	0.347	0.014	<0.001	0.288	0.014	<0.001
Outgroup vs stranger	-0.269	0.014	<0.001	-0.171	0.014	<0.001

In the second alternative approach, we ran all possible regressions with investments in attack and defence predicted by the variable ingroup vs outgroup$_x$ and stranger, with x being one of the 50 outgroup countries. We ran these regressions for all the 51 countries, giving a total of 2,550 regressions. Then, we compared the results of these regressions with the distribution of outputs of the same number of regressions where we randomly allocated subjects to the variable ingroup or outgroup/stranger (also known as permutation tests; shown on the left panels in Fig S3). As shown, the patterns between the random allocation (left) and our actual treatment (right) look quite different, with our actual treatment showing most of the estimates above 0 (indicating the presence of the nasty neighbour effect), and with most contrasts to be significant at 5% level (blue dots). Results do not meaningfully change if we only consider ingroup vs outgroup$_x$ differences (Fig S4).

Fig S3. **Distribution of significant p-values for attack (top panels) and defence (bottom panels) investments.** Plot showing the direction and frequency of significant estimates for the ingroup vs outgroup/stranger dummy predicting attack investments, considering each country independently as a potential outgroup. On the left, distribution of the estimates when we randomly assign decisions into the ingroup or outgroup/stranger dummy variable. On the right, the actual distribution of the estimates is shown.

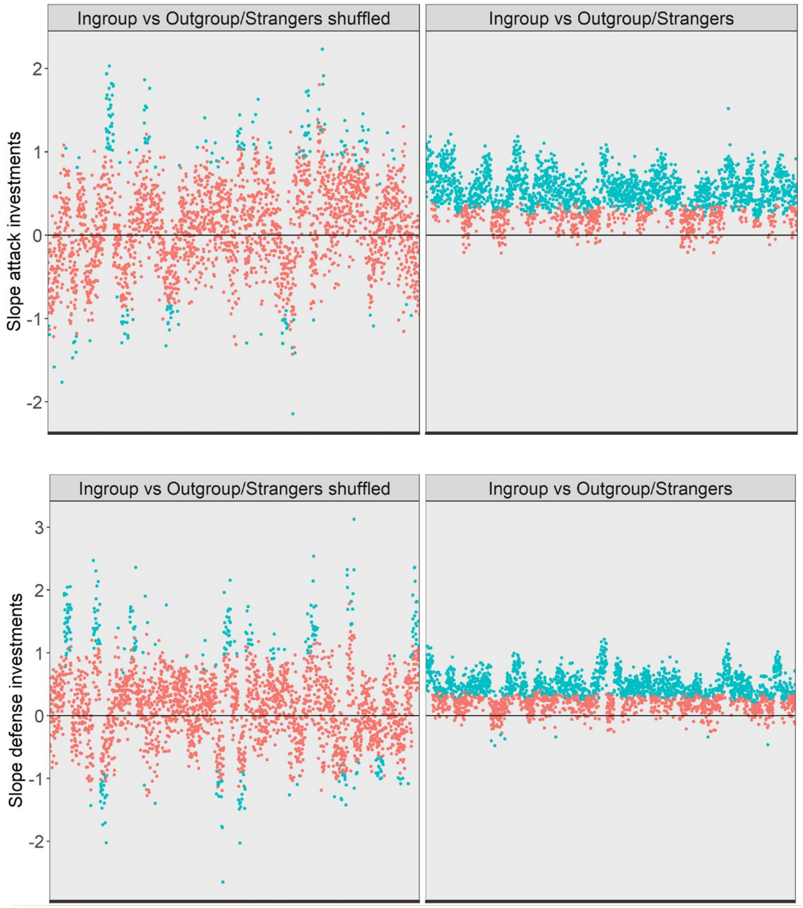

Fig S4. **Distribution of significant p-values for attack (top panels) and defence (bottom panels) investments.** Plot showing the direction and frequency of significant estimates for the ingroup vs outgroup dummy predicting attack investments, considering each country independently as a potential outgroup. On the left, distribution of the estimates when we randomly assign decisions into the ingroup or outgroup dummy variable. On the right, the actual distribution of the estimates is shown.

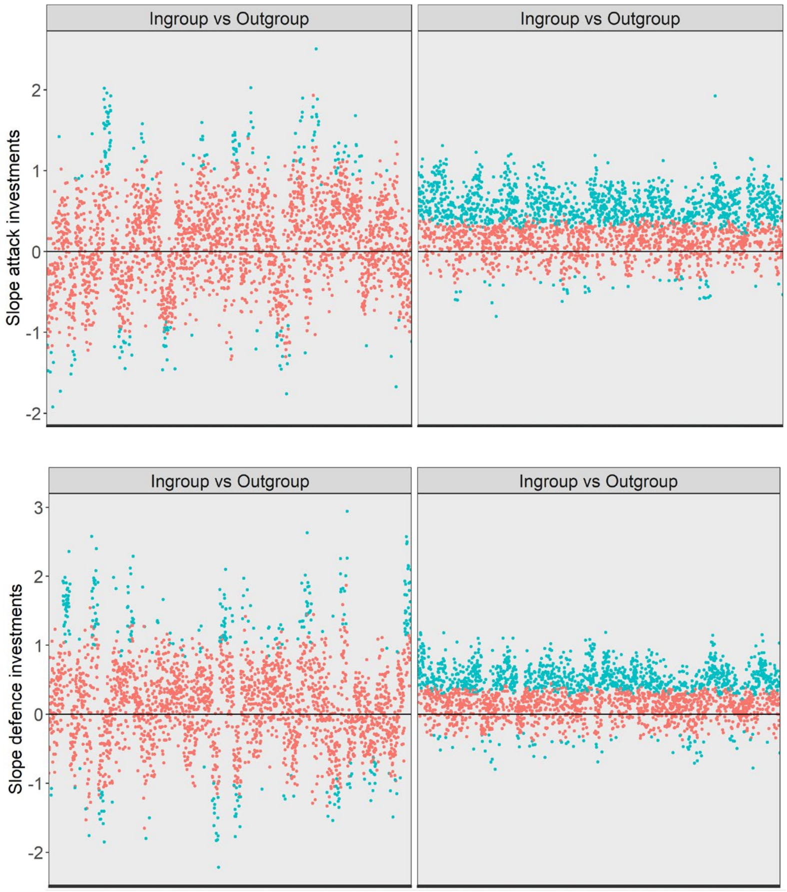

Finally, in Table S4 we control for several factors that could affect the extent to which people invested in attack and defence toward ingroup members (vs outgroup members and strangers). We find that the main predictor remains significant: people invest more in competing with opponents from the same country, compared to opponents from outgroup countries and strangers. Overall, we find that the effect is independent of age, gender, and education (i.e., lack of significant interaction effects). Only for defence, we find a significant interaction between gender and the extent to which people discriminate between ingroup members and outgroup members/strangers. The difference in attack between ingroup vs outgroup/stranger is larger for women compared to men (but still significant for both men and women). In line with results reported in previous research (*78*), men invested more resources in attack and defence than women, older people invested less than younger people, and people with higher education invested more in conflict than people with lower education.

Table S4 Mixed-effect model of ingroup (vs outgroup & stranger) with controls predicting investment decisions. Tests are two-sided. $N_{\text{observations}}$ = 362,365; N_{subjects} = 12,833, $N_{\text{countries}}$= 51. The variable age is standardized.

	Attack			Defence		
	b	***SE***	***p***	***b***	***SE***	***p***
Ingroup vs outgroup & stranger	0.283	0.06	<0.001	0.248	0.058	<0.001
Gender	-0.312	0.035	<0.001	-0.260	0.035	<0.001
Age	-0.088	0.018	<0.001	-0.116	0.018	0.001
Education	0.084	0.017	<0.001	0.085	0.016	<0.001
Ingroup vs outgroup & stranger × gender	0.079	0.029	0.007	0.052	0.029	0.071
Ingroup vs outgroup & stranger × age	0.009	0.015	0.533	-0.002	0.014	0.533
Ingroup vs outgroup & stranger × education	0.005	0.013	0.702	0.002	0.013	0.702

2.1.2. Cross-societal models. As already suggested by the little variation shown in Fig. 1b (Main Text), we find no evidence that greater investments in conflict with ingroup members (vs outgroup members and strangers) between countries are associated with prominent cross-societal factors (*12*, *24*) (globalization, GDP per capita, rule of law, government effectiveness, historical prevalence of pathogens, and patriotism, etc.) (Figure S3). Only GDP per capita is significantly associated with differences in attack investments between ingroup and outgroup & stranger (r = 0.29, p = 0.04).

Yet, we do find stronger variation in how people invested in within-group aggression between countries; that can also account for the nasty neighbour effect between countries. In particular, we found that between-country differences in aggression and defence toward ingroup members are negatively associated with wealth, quality of institutions, and egalitarian values and positively associated with hierarchical values. While these results are only exploratory, they fit with the insights on the potential mechanisms associated with the nasty neighbour effect in Study 2 to 5.

Figure S5 Bivariate correlations between prominent cross-societal factors and the nasty neighbour effect across societies (cells with an × indicate non-significant associations).

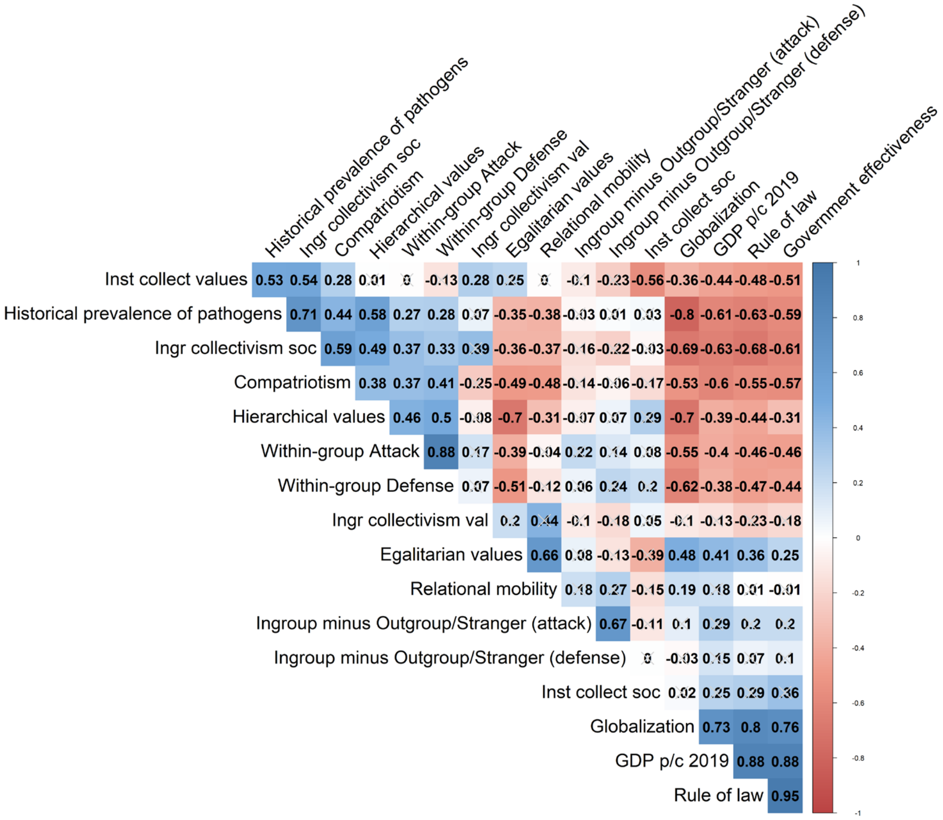

2.1.3. Geographical and cultural distance. In this section, we report the results on geographical and cultural distance. Such analyses can provide additional support that people are more competitive toward ingroup than outgroup members and strangers. Geographical bilateral distances measure city-level data to account for the geographic distribution of population inside each nation (*41*). Geographical distance is available for 225 countries and consists of the distance between two countries based on bilateral distances between the biggest cities of those two countries. Socio-psychological distance (or cultural distance) is a measure of the overall psychological differences between societies. To build this index, we retrieved bilateral cultural distances data from http://culturaldistance.com/ (all dimensions) or here. This indicator is calculated from data on beliefs, values and behaviours that people have about or associate with their own nation retrieved from the World Values Survey (two waves: 2005-2009; 2010-2014; for a complete report of the analytic strategy to calculate this indicator see (*42*)). The World Values Survey dataset is composed of 170,247 participants from 80 nations (which altogether covers 85% of the world population). The list of measures used to calculate the socio-psychological distances can be found here: https://michael.muthukrishna.com/cultural-distance-data/.

Table S5 shows results from independent models of geographical and cultural distance predicting attack and defence decisions, and geographical and cultural distance in the same model with GDP per capita as a control. Across independent models, and models controlling for GDP, results remain the same: people invest more resources in attack and defence with opponent from geographically or culturally closer countries, compared to opponents from more distant countries. Moreover, results remain robust even when removing the most distant countries (i.e., removing countries above the third quartile of geographical distance; attack: $b = -0.096$, $p < .001$, defence: $b = -0.084$, $p < .001$). That said, it is important to note that, although the effect of geographical distance seems robust, the study was not specifically designed to test for geographical distance and future research is warranted to more precisely test the relation between distance and competition.

Table S5 Mixed-effect model of distance on investment decisions. Tests are two-sided. For geographical distance: $N_{observations} = 330{,}926$, $N_{subjects} = 12{,}605$, $N_{countries} = 50$; For cultural distance: $N_{observations} = 216{,}138$; $N_{subjects} = 10{,}345$, $N_{countries} = 41$. Models controlling for GDP (bottom three rows): $N_{observations} = 205{,}652$, $N_{subjects} = 10{,}087$, $N_{countries} = 40$.

	Investment in Attack			Investment in Defence		
Contrast	*b*	*SE*	*p*	*b*	*SE*	*p*
Geographical distance	-0.066	0.003	<0.001	-0.063	0.003	<0.001
Cultural distance	-0.039	0.004	<0.001	-0.023	0.004	<0.001
Controlling for GDP						
Geographical distance	-0.037	0.004	<0.001	-0.035	0.004	<0.001
Cultural distance	-0.034	0.003	<0.001	-0.017	0.004	<0.001
GDP per capita 2019	0.171	0.004	<0.001	0.148	0.003	<0.001

2.1.4. Instructions

In this section, we include the English version of the instructions of the attacker-defender game. Written instructions were distributed across several pages and followed by pictures to aid the understanding of the game.

2.1.4.1. Instruction of the attacker-defender game

Welcome to the study.

This is a study about decision making.

The study involves participants from 51 countries around the world.

You will be asked to make decisions in several decision making tasks.

You will be paired with a different person in each decision making task.

Algeria, Argentina, Australia, Austria, Belgium, Brazil, Bulgaria, Canada, Chile, China, Colombia, Czech Republic, Egypt, Finland, France, Germany, Greece, Hong Kong, Hungary, India, Indonesia, Ireland, Israel, Italy, Japan, Kenya, Malaysia, Mexico, Morocco, The Netherlands, Nigeria, Peru, Poland, Portugal, Romania, Russia, Saudi Arabia, Singapore, South Africa, South Korea, Spain, Sweden, Switzerland, Taiwan, Thailand, Tunisia, Turkey, United Arab Emirates, United Kingdom, United States, Vietnam.

Please read the instructions carefully because you have the possibility to earn Monetary Units based on your decisions and others' decisions.

Each Monetary Unit (MU) is worth ## when making decisions.

You will be asked to make decisions in several decision making tasks.

You will be paired with a different person in each decision making task.

In this task, you will make decisions in two roles : (1) PERSON A, (2) PERSON B.

Both PERSON A and PERSON B will receive 10 Monetary Units.

Each monetary unit is #. When you interact with a person from a different country, then the value of an MU will be based on the average amount of pay for 2.5 minutes of work in that country. So, an MU has the same value across all of the countries participating in this study.

PERSON A: decides whether to challenge PERSON B to take his or her MU.

PERSON B: decides whether to prevent PERSON A from taking his or her MU.

PERSON A can challenge PERSON B by investing between 0 and 10 MU to the challenge pool (5 in this example).

PERSON B gets to decide whether to invest between 0 and 10 MU to prevent PERSON A from taking his or her MU (2 MU in this example).

The MU invested to challenge PERSON B are lost.

However, if they surpass the MU PERSON B has assigned to stop the Challenge, PERSON A receives the MU of PERSON B that were not assigned to the Challenge pool.

Hence, in this example, PERSON A will keep the 5 MU that PERSON A did not invest in the challenge pool plus 8 MU from PERSON B (PERSON B's remaining endowment: 10 MU - 2 MU = 8 MU). Hence, PERSON B will lose all of his or her MU because PERSON B's investment in prevention was less than PERSON A's investment in challenge.

However, if PERSON B assigns the same MU as PERSON A or more (6 in this example), PERSON A does not receive the remaining MU from PERSON B.

In this case, PERSON A and PERSON B keep whatever they have left of their starting capital (in this example, 5 MU for PERSON A, and 4 MU for PERSON B).

In this study, you and the other participants will be asked to make several decisions either as PERSON A or PERSON B.

Each decision will be made with a partner from one of the countries participating in this study.

At the end of this study, we will randomly select one of your decisions, match this decision with a corresponding partner participating in this study, and pay you and the other person accordingly.

If you are selected to be PERSON A, you will be paired with a person that has been selected as a PERSON B and vice versa.

2.1.4.2 Trial rounds.

To make sure you have understood the instructions, please calculate the final earnings for the following, hypothetical scenario:

Remember: Both PERSON A and PERSON B begin the task with 10 MU

You are PERSON A and decide to invest 4 MU to take MU from PERSON B. PERSON B invests 8 MU to prevent PERSON A (YOU) from taking your MU. Then, (choose one)

YOU earn 4, PERSON B earns 8

YOU earn 8, PERSON B earns 0

YOU earn 6, PERSON B earns 2

YOU earn 14, PERSON B earns 6

YOU earn 16, PERSON B earns 0

To make sure you have understood the instructions, please calculate the final earnings for the following, hypothetical scenario:

Remember: Both PERSON A and PERSON B begin the task with 10 MU

You are PERSON B and decide to invest 1 MU to prevent PERSON A from taking your MU. PERSON A invests 3 MU to take MU from PERSON B (YOU).
Then, (choose one)
YOU earn 0, PERSON A earns 7
YOU earn 1, PERSON A earns 3
YOU earn 9, PERSON A earns 7
YOU earn 0, PERSON A earns 16
YOU earn 16, PERSON A earns 0

2.1.4.3 Decision blocks.
(if participant was in the role of Person A)
PLEASE NOTE: IN THE NEXT BLOCK YOU WILL BE ASKED TO MAKE DECISIONS IN THE ROLE OF:
PERSON A

PERSON A can challenge PERSON B by investing between 0 and 10 MU to the challenge pool.
Each decision will be made with a partner from one of the countries participating in this study.
In the picture below, you can see an example of decision with a person from United States.
Please click below to start.

(if participant was in the role of Person B)
PLEASE NOTE: IN THE NEXT BLOCK YOU WILL BE ASKED TO MAKE DECISIONS IN THE ROLE OF:
PERSON B
PERSON B gets to decide whether to invest between 0 and 10 MU to prevent PERSON A from taking his or her MU.
Each decision will be made with a partner from one of the countries participating in this study.
In the picture below, you can see an example of decision with a person from United States.

Please click below to start.

2.1.4.3. Example decision stage attack and defence with ingroup members (Australia)

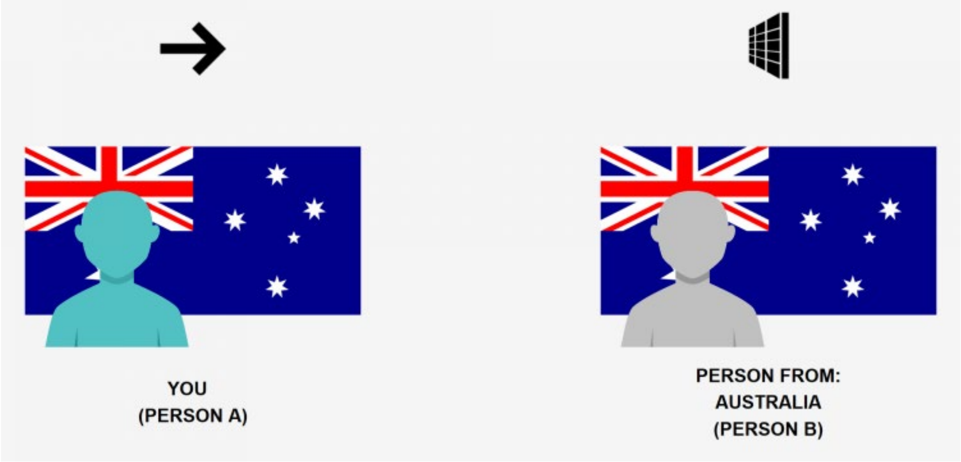

Number of MU you invest to take MU from PERSON B

Each Monetary Unit (MU) is ##

Set the button to a number

PLEASE NOTE: in the next page, you will make a decision with a different partner

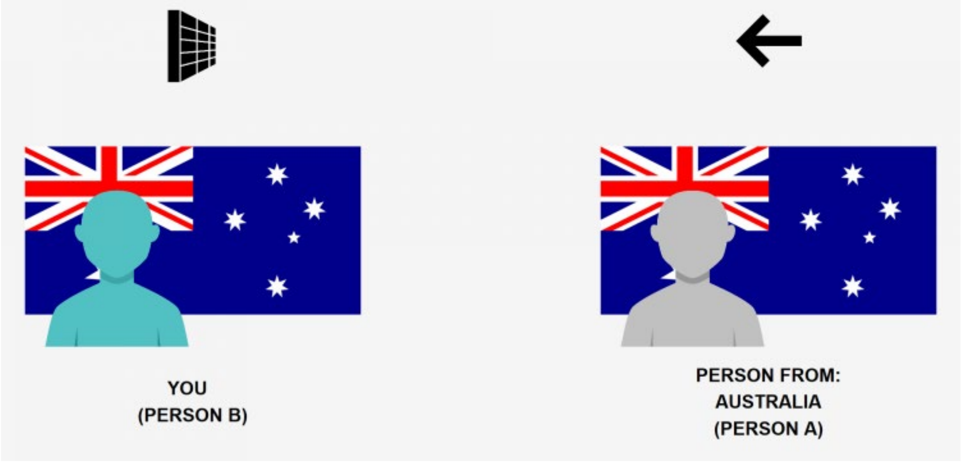

Number of MU you invest to prevent PERSON A from taking your MU

Each Monetary Unit (MU) is 1 ####

Set the button to a number

2.1.4.4. Example picture attack and defence with outgroup members

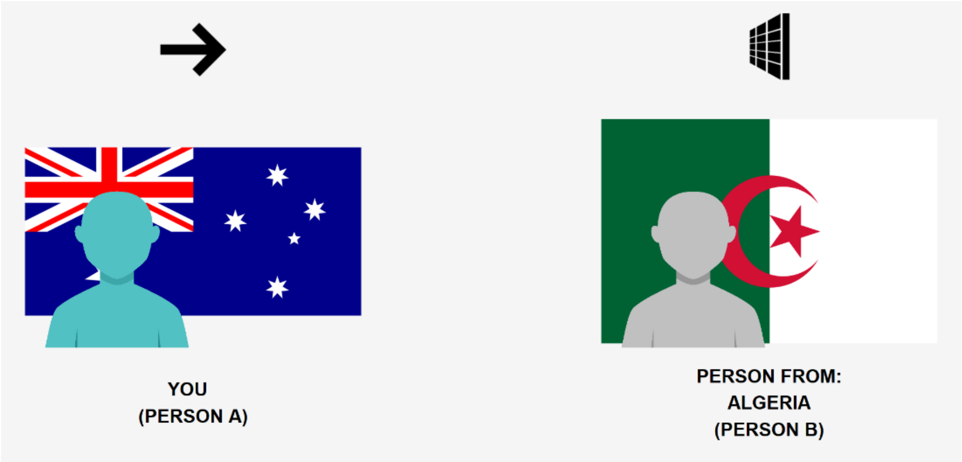

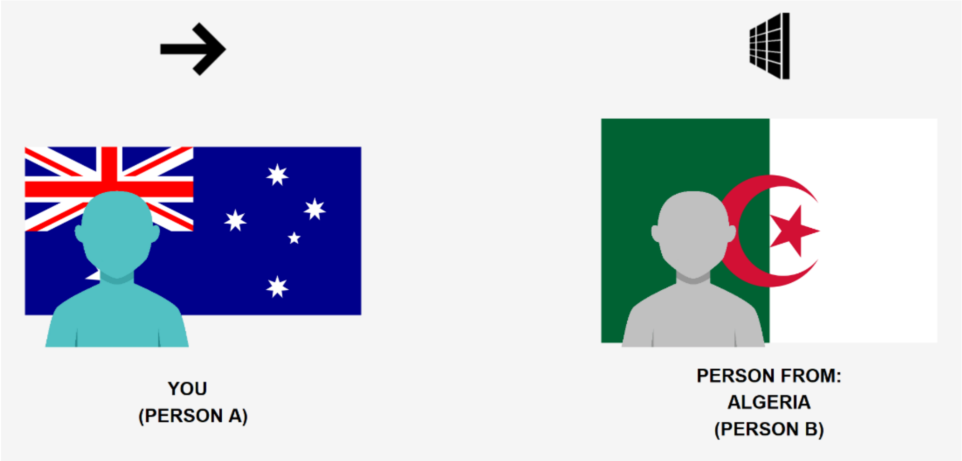

2.1.4.5. Example picture attack and defence with unknown opponent

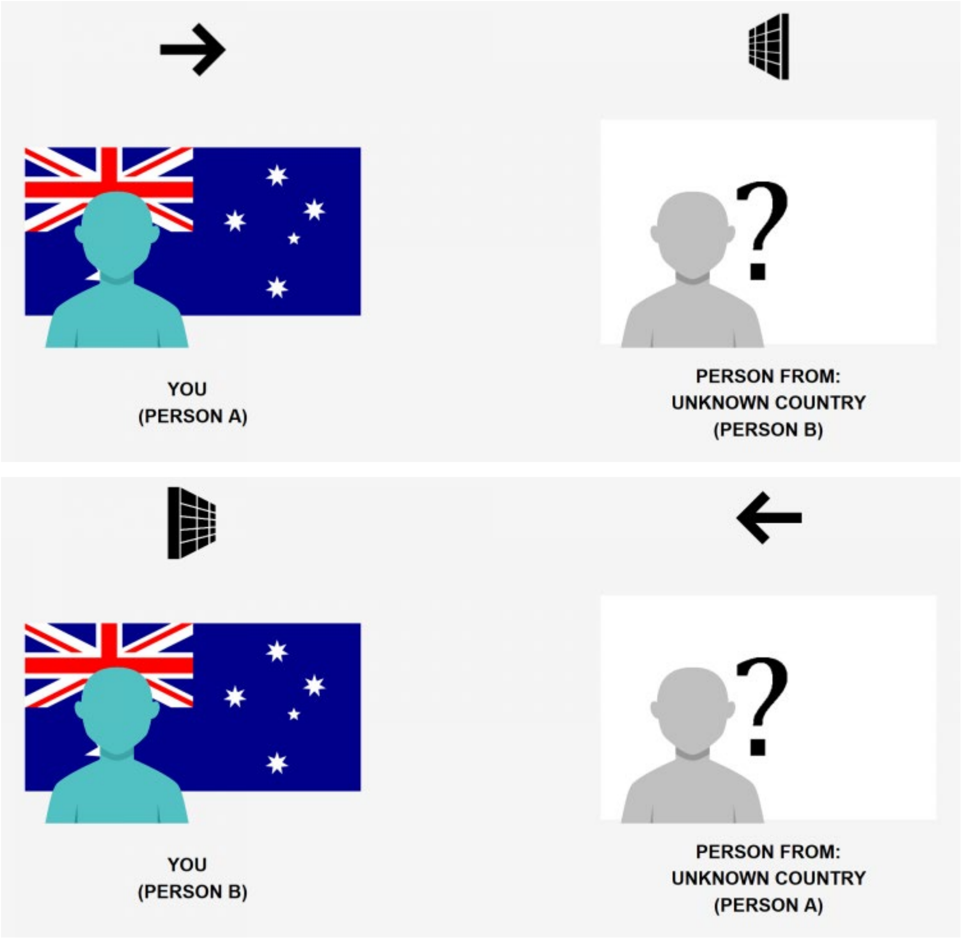

2.2. Study 2

Participants were recruited through the Busara Center for Behavioral Economics. Groups were selected based on the prevalence of these communities in Nairobi (Kikuyu, Luo, Kamba and Luhya).

2.2.1. Treatment effects. Table S6 shows results from models of the ingroup (vs outgroup & strangers) contrast predicting attack and defence decisions. We excluded participants that failed simple attention checks in the lab (~27%). We also control for the community pairing (see Methods). Again, we find no support for the parochial competition hypothesis. In line with the cross-cultural study, people invested more resources in defending against ingroup members, compared to outgroup members and strangers. When we explore whether investments in attack or defence towards outgroup members differ from investments toward strangers we find, contrary to the cross-cultural Study 1, no significant differences in attack and defence with opponents from outgroup communities, compared to unidentified strangers. The exclusions do not meaningfully affect the overall interpretation of the results: Participants still invest more in attacking and defending from ingroup than outgroup members and strangers, yet the effect of defence investments becomes not significant.

Table S6 Mixed-effect model of treatment effects on investment decisions. Tests are two-sided. $N_{\text{observations}}$ = 1,656; N_{subjects} = 552. Community pair: 1 = Luhya and Kamba; 0 = Kikuyu and Luo. For outgroup vs stranger contrast: $N_{\text{observations}}$ = 1,104; N_{subjects} = 552. Outgroup = 0, Stranger = 1.

	Investment in Attack			Investment in Defence		
Contrast	*b*	*SE*	*p*	*b*	*SE*	*p*
Ingroup vs outgroup & stranger	0.046	0.085	0.586	0.178	0.085	0.036
Pair (Kikuyu/Luo; Luhya/Kamba)	-0.019	0.184	0.915	0.002	0.182	0.990
Outgroup vs stranger	-0.029	5.940	0.996	-6.379	5.779	0.270

2.3. Study 3

2.3.1. Treatment effects. Table S7 shows results from models of the ingroup (vs outgroup & strangers) contrast predicting attack and defence decisions, and models with geographical and cultural distance predicting attack and defence investments. Since a very small fraction of participants failed to respond to our attention check (<3%), all participants were included in the analyses. Excluding participants who did not pass the attention check did not affect the results. In line with Study 1, we find that individuals invest more resources in both attack and defence towards ingroup members, compared to outgroup members and strangers. Moreover, as in Study 2, we find no significant differences in both attack and defence with opponents from outgroup countries compared to unidentified strangers. Finally, and in line with Study 1, we find that geographical and cultural distance are negatively associated with conflict, suggesting that people invest more resources in attack and defence when interacting with opponents from geographically and culturally closer countries than more distant countries (Table S8). Altogether, these results replicate and support the conclusions from our first two studies.

Table S7 Mixed-effect model of treatment effects on investment decisions. Tests are two-sided. $N_{observations}$ = 363,231; $N_{observations}$ = 10,827; $N_{subjects}$ = 401. Ingroup = 0, Outgroup & stranger = 1.

	Investment in Attack			Investment in Defence		
Contrast	*b*	*SE*	*p*	*b*	*SE*	*p*
Ingroup vs outgroup & stranger	0.285	0.073	<0.001	0.198	0.069	0.007
Outgroup vs stranger	0.136	0.073	0.062	-0.061	0.068	0.372

Table S8 Mixed-effect model of distance on investment decisions. Tests are two-sided with $N_{observations}$ = 10,030; $N_{subjects}$ = 401. For cultural distance: $N_{observations}$ = 8,466; $N_{subjects}$ = 401.

	Investment in Attack			Investment in Defence		
Contrast	*b*	*SE*	*p*	*b*	*SE*	*p*
Geographical distance	-0.036	0.014	0.013	-0.022	0.014	0.099
Cultural distance	-1.114	0.178	<0.001	-0.54	0.169	0.001

3. The nasty neighbour effect emerges independent of ingroup favouritism

3.1. Study 4

3.1.1. Treatment effects. Table S9 shows the results from mixed effect models of decisions in the trust game and in the attacker-defender contest. In line with Study 3, all participants were included in the analyses. Only one participant did not pass the attention check. Excluding the participant who did not pass the attention check did not affect the results. We see that people trust ingroup members more than outgroup members and strangers, and return more resources when paired with ingroup members, compared to outgroup members and strangers. We also find, as in Study 1-3, that people invest more resources in attacking ingroup members than outgroup members and strangers. We do not replicate this treatment effect on defence investments, although the direction of the effect is in line with the nasty neighbour effect.

Table S9 Mixed-effect model of treatment effects on investment decisions. Tests are two-sided. $N_{observations}$ = 4,800; $N_{subjects}$ = 300. Ingroup = 0, Outgroup & stranger = 1.

	Trust			Trustworthiness		
Contrast	*b*	*SE*	*p*	*b*	*SE*	*p*
Ingroup vs outgroup & stranger	0.327	0.046	<0.001	2.330	0.501	<0.001
Outgroup vs stranger	-0.131	0.045	0.003	-1.536	0.498	0.002

	Attack			Defence		
Contrast	*b*	*SE*	*p*	*b*	*SE*	*p*
Ingroup vs outgroup & stranger	0.236	0.079	0.003	0.071	0.070	0.311
Outgroup vs stranger	0.104	0.078	0.189	-0.039	0.069	0.578

3.1.2. Association between ingroup favouritism and nastiness. In this section, we report the analyses regarding the relation between ingroup favouritism in the trust game and the nasty neighbour effect in the attacker-defender contest game. To do so, we calculated the difference between trust (return) toward the own country and trust (return) toward others and averaged these two indicators into one overall indicator of ingroup favouritism in cooperation. Likewise, we calculated the difference between attack (defence) towards the own country vs others and created a measure of overall (ingroup) nastiness by averaging nastiness in attack and defence. We found no evidence that ingroup favouritism and (ingroup) nastiness are correlated ($t(298) = 0.392$, $p = 0.695$).

Second, we show the relation between the aspects of ingroup favouritism and (ingroup) nastiness that are conceptually related (Main Text, Fig 2b and 2c). Whereas ingroup favouritism in trust and ingroup

favouritism in return were positively and significantly associated (r = 0.425, $p < .001$) as well as nastiness in attack and defence (r = 0.33, $p < .001$), there is a positive but insignificant correlation between (ingroup) nastiness in defence and ingroup favouritism trust (r = 0.097, p = .093; Fig. 2, Main Text) and a positive yet insignificant correlation between nastiness in attack and parochial returns (r = 0.10, p = .082; Fig 2, Main Text). If parochial cooperation is the flip side of parochial competition, we should have seen negative (and arguably more substantial) correlations.

3.2. Re-analysis cross-cultural study from Romano et al. (2021)

In this section, we report a re-analyses of a cross-cultural dataset that contains data on national ingroup favouritism in cooperation in the prisoner's dilemma (*12*). In this cross-cultural study (N = 18,411, sample stratified by age, gender, and income), participants across 42 societies made several independent decisions to cooperate in a prisoner's dilemma with ingroup members (same nation), outgroup members (operationalized by an opponent extracted from a pool of 16 outgroup nations), and an unidentified stranger. In this study, the authors found that, on average, people cooperated more with ingroup members, compared to outgroup members and strangers.

Yet, as results from Study 4 suggest that ingroup favouritism in cooperation and neighbour nastiness are independent behavioural strategies, we can expect substantial individual heterogeneity, with people being nasty neighbours even in situations where ingroup favouritism cooperation is typically observed. To run this individual heterogeneity analysis, we divided participants in three types. *Ingroup favouring* types were defined as people that invested more resources in cooperating with ingroup members, compared to outgroup members and strangers. *Nasty neighbours* types were defined as people that invested more resources in cooperating with outgroup members and strangers, than ingroup members. *Neither ingroup favouring nor nasty* types were defined as people that did not discriminate between ingroup, outgroup members, and strangers.

Overall, we found that 8577 individuals (48%) could be categorized as ingroup favouring, 5,736 individuals (32%) could be categorized as nasty neighbours, and 3174 individuals (20%) could be categorized as neither ingroup favouring nor nasty. The difference in proportion of types was statistically significant, $X^2(2)$ = 1986.4, $p < .001$. A pair-wise chi-square test, testing whether the proportion of nasty neighbours types was higher than the 'neither ingroup favouring nor nasty' types, was also significant ($p < .001$). In sum, results from the re-analysis reveal that (*i*) neighbour nastiness can also be observed in other experimental paradigms, such as the prisoner's dilemma game and that (ii) people can be classified as nasty neighbour or ingroup favouring in the same situation.

4. Within-group status and resource competition can turn humans into nasty neighbours

4.1. Moderators and mediators in Study 3

Table S10 shows results from independent interaction models between a moderator variable (see *Methods*) and the ingroup (vs outgroup & strangers) variable. In the previous analyses we did not find substantial differences in the observed patterns between attack and defence investments, and for the current analyses aggregated across both attack and defence. The only significant interaction in the model is perceived status within a group; people that self-report to have lower status in their nation are the ones that discriminate more in their conflict investments between ingroup vs outgroup members and strangers (also see Fig. 3a, Main Text).

Table S10. Independent mixed-effect models predicting conflict decisions (attack & defence combined). Tests are two-sided. $N_{\text{observations}} = 21{,}654$; $N_{\text{subjects}} = 401$.

Investments in conflict (attack and defence)	*b*	*SE*	*p*
Ingroup (vs outgroup/strangers) × Perceived status ingroup	0.188	0.077	0.015
Ingroup (vs outgroup/strangers) × Perceived status outgroup	0.091	0.079	0.251
Ingroup (vs outgroup/strangers) × Perceived financial scarcity	0.011	0.04	0.788
Ingroup (vs outgroup/strangers) × Reputational concern ingroup	0.018	0.039	0.634
Ingroup (vs outgroup/strangers) × Reputational concern outgroup	-0.056	0.039	0.152
Ingroup (vs outgroup/strangers) × Generosity (qualitative item)	-0.029	0.025	0.249
Ingroup (vs outgroup/strangers) × Generosity (quantitative item)	0.001	0.001	0.474
Ingroup (vs outgroup/strangers) × Risk preferences	-0.031	0.026	0.243
Ingroup (vs outgroup/strangers) × Identification with nation	-0.001	0.04	0.979

We also explored the role of potential mediators: expectations, perceived competition over scarce resources and perceived similarity. We assessed these measures for each country participating in the study and ran multilevel mediation models (with subjects as random intercepts) using the causal mediation package (*86*). While we find that all three measures can account for the effect of national membership on conflict (Table S11), perceived competition over scarce resources is the only variable that fully mediates this association and that explains the highest proportion of variance. Perceived competition over scarce resources was assessed by the following question: “Competition plays an important role within and across countries. At a global and local stage, individuals, companies, and governments compete for scarce resources, like access to natural resources, access to new technology, participation in the labour market, or transnational agreements that govern the rights and obligations of citizens from different countries. Such competition can directly or indirectly also influence your own well-being. Please rate below, how much you think your own well-being is influenced by competition with people from different countries (0 = not at all, 10 = very much)”. Participants responded for all opponent countries, including their own country. The model with

perceived competition over scarce resources as mediator and the other variables as controls is the only model that fully explains the nasty neighbour effect and the only one that explains a significant proportion of mediation in a multi-level mediation model (Table S11). Neither perceived similarity nor expectations explain a significant proportion of the mediation, when controlling for the other mechanisms. Moreover, we also ran models that included interactions between beliefs, perceived competition, and similarity with group membership predicting conflict expenditure in the attacker defender game. The conclusions do not change if we treat all variables as moderators: only perceived ingroup status and perceived competition over scarce resources show a significant interaction predicting conflict investments towards ingroup (vs outgroup & stranger) members (i.e., expectations and similarity do not significantly interact with the nasty neighbour effect in the attacker-defender game). In summary, we find that perceived competition over scarce resources fully mediates the effect of national membership (ingroup vs outgroup/strangers) on conflict. This result sheds light on the potential mechanisms related to the nasty neighbour effect we observed.

Table S11. Multi-level mediation models.

	b	*95% CI (LL; UL)*	*p*
**Expectations**			
Indirect effect	-0.241	-0.033; -0.02	<0.001
Direct effect	-0.226	-0.334; -0.11	<0.001
Total effect	-0.250	-0.357; -0.14	<0.001
Proportion mediated	0.094	0.060; 0.22	<0.001
**Perceived Similarity**			
Indirect effect	-0.065	-0.110; -0.03	<0.001
Direct effect	-0.182	-0.294; 0.05	<0.001
Total effect	-0.247	-0.359; -0.12	<0.001
Proportion mediated	0.264	0.109; 0.64	<0.001
**Perceived Competition**			
Indirect effect	-0.234	-0.266; -0.20	<0.001
Direct effect	-0.014	-0.157; 0.10	0.78
Total effect	-0.248	-0.382; -0.13	<0.001
Proportion mediated	0.943	0.594; 1.70	<0.001
**Perceived Competition** (controlling for perceived similarity and expectations)			
Indirect effect	-0.230	-0.263; -0.20	<0.001
Direct effect	-0.021	-0.163; 0.13	0.66
Total effect	-0.252	-0.383; -0.10	<0.001
Proportion mediated	0.911	0.581; 2.24	<0.001
**Expectations** (controlling for perceived similarity and competition)			
Indirect effect	-0.018	-0.027; -0.01	<0.001
Direct effect	-0.018	-0.129; 0.10	0.82
Total effect	-0.036	-0.147; 0.08	0.64
Proportion mediated	0.176	-2.445; 3.17	0.64
**Perceived Similarity** (controlling for perceived competition and expectations)			
Indirect effect	-0.020	-0.023; -0.01	0.26
Direct effect	-0.022	-0.148; 0.10	0.74
Total effect	-0.042	-0.170; 0.08	0.94
Proportion mediated	0.173	-1.830; 3.97	1.00

4.2. National identity and perceived competition in Study 4

Table S12 shows how observed ingroup favouritism in the trust game and (ingroup) nastiness in the attacker-defender game were predicted by different psychological mechanisms. In line with research on ingroup favouritism, social identification was significantly associated with ingroup favouritism in trust and trustworthiness but was not related to the nasty neighbour effect in attack and defence. In contrast, differences in perceived competition toward ingroup vs outgroup members were significantly associated with the nasty neighbour effect in attack and defence but not with ingroup favouritism in trust and trustworthiness.

Table S12. Independent regression models predicting ingroup favouritism in the trust game or the nasty neighbour effect in the attacker-defender game.

	b	***SE***	***p***
Social identification predicting ingroup favouritism in trust	0.131	0.031	<0.001
Social identification predicting ingroup favouritism return	0.844	0.326	0.010
Social identification predicting nasty attack	-0.001	0.052	0.971
Social identification predicting nasty defence	0.001	0.047	0.999
Perc. competition ingroup minus outgroup predicting parochial trust	-0.012	0.019	0.527
Perc. competition ingroup minus outgroup predicting parochial return	-0.032	0.021	0.875
Perc. competition ingroup minus outgroup predicting nasty attack	0.069	0.032	0.035
Perc. competition ingroup minus outgroup predicting nasty defence	0.052	0.029	0.073

4.3. Study 5

In Study 5, our aim was to test whether we could observe the nasty neighbour effect in minimal groups by experimentally manipulating two mechanisms that emerged to be associated with the nasty neighbour effect: resource scarcity and status concerns. All participants were included in the analyses. Only one participant did not pass the attention check. Excluding the participant who did not pass the attention check did not affect the results.

4.3.1. Within vs between group competition. We introduced two competitive treatments. In the *between-group competition* conditions (present vs absent), we expected people to become more parochial once the opportunity to compete for a bonus in Stage 1 was introduced (see *Methods*). Our treatment was successful in manipulating perceived competition with ingroup vs outgroup members, as people perceived a relative higher competition with outgroups compared to ingroup members when between-group competition was present (vs absent: Welch two-sample t-test, $t(472.66) = -4.475$, $p < 0.001$). Moreover, in line with our preregistered hypothesis, we find that people were more parochial (i.e., higher investments towards the group-exclusive club pool) when between-group competition was present, compared to when between group competition was absent (paired t-test, $t(275) = 5.773$, $p < 0.001$). In the between-group competition conditions, people also invested more resources to the parochial compared to the universal pool (paired t-test, $t(275) = 4.694$, $p < 0.001$).

In Study 5, we also aimed to implement within-group competition by adding the possibility to get a bonus in Stage 2. In the within-group competition present condition, people were informed that they would get a bonus if, at the end of the experiment, they would earn more than their group members (in the control condition, the bonus was randomly allocated). In line with Stage 1, the value of the bonus was relatively low (in stage 2 participants are endowed with 20 MU and could use 5 to punish). Hence, the payoff maximizing strategy would be to keep in Stage 1 and not punish in Stage 2. Contrary to the between-group competition condition, this treatment was not successful in increasing the relative perceived competition with ingroup vs outgroup members (Welch two-sample t-test, $t(543.57) = -1.201$, $p = 0.231$). We suspect that this failure to implement within-group competition (vs not) may be due to the competitive nature of Stage 1, some misunderstanding among participants about the allocation of the bonus in Stage 2, or the relatively low incentive to get an extra bonus. Indeed, we find that the within-group competition treatment was not significantly associated with the nasty neighbour effect in punishment (linear regression, $b = -0.034$, $p = 0.489$). In contrast, the nasty neighbour effect was observed among people who perceived higher competition with ingroup relative to outgroup members, regardless of the treatment (linear regression, $b = 0.048$, $p < 0.001$). Moreover, in our setting, high status more likely result by being selfish in Stage 1, while low status more likely result by being a co-operator in Stage 1. While we believe the possibility for this

confound to be unlikely (because participants were not provided feedback after Stage 1), future research is needed to study the interplay between status and ingroup aggression in other settings.

4.3.2. Status differences. In Stage 2, we manipulated the earning status of the target of attack. Participants could assign up to 5 MU as ‘deduction points’ to an ingroup member and to an outgroup member. Deductions reduced the target’s earnings at a 1 to 3 ratio. We operationalized member status in terms of their earnings relative to others in their group and in the outgroup, and elicited attack decisions in 11 possible scenarios. In five scenarios, the participant had lower earning status, in one they had equal status, and in five they had greater earning status. Looking at the general attack decisions by status, we found that, in general, people tend to attack more individuals with higher status (than lower status), and that was true for both ingroup ($b = 0.30$, $p < .001$) and outgroup members ($b = 0.66$, $p < .001$).

Across all conditions, we find a significant interaction between status of the target and group membership (see Table S13). In particular, from the Johnson Neymann plot shown in Figure S6, people were parochial in their attack (more attack to outgroup vs ingroup members) when the targets of their attack were high earning status individuals and were nasty neighbours (more attack to ingroup vs outgroup members) when the target of their punishment were low earning status individuals. In this study, we also included measures of beliefs for Stage 1 and 2. When replacing behaviour with beliefs, the results for both Stage 1 and 2 remain the same.

Table S13. Independent mixed effect models with subjects as random intercept and the interaction between status of the target and group membership predicting punishment. Tests are two-sided with $N = 552$. Results remain robust when applying Bonferroni correction for multiple hypotheses testing.

	b	***SE***	***p***
Within competition No / Between competition No	0.256	0.037	<0.001
Within competition No / Between competition Yes	0.463	0.046	<0.001
Within competition Yes / Between competition No	0.323	0.045	<0.001
Within competition Yes / Between competition Yes	0.368	0.048	<0.001
Across all treatments	0.353	0.022	<0.001

Figure S6. Status differences favour the emergence of within-group nastiness. Floodlight plot showing the regions of differences in status of the target of attack (*x* axis, standardized) for which the effect of ingroup vs outgroup (*y* axis) on attack becomes significant. The vertical lines in the floodlight plot show the exact values at which significance begins and ends. Blue lines indicate significance at 5% level.

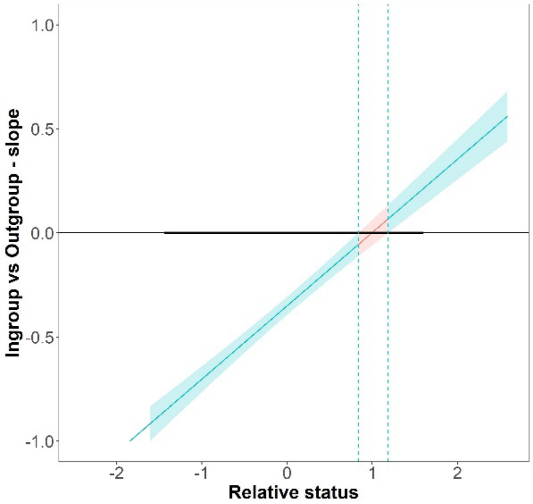

5. Complementary study

Next to the experiments reported in the Main Text we performed an additional study to examine the possibility that ingroup favouritism or neighbour nastiness emerges also in preferences for whom to compete with (*87*). The literature on ingroup favouritism suggests that when given a choice, individuals prefer to compete with those in outgroups, rather than within their own group. The nasty neighbour effect identified in our main experiments may counter-act or even reverse this preference. A secondary aim of this complementary study was to examine whether and how preferences for competing within or between groups relate to ingroup favouritism in dictator giving.

5.1. Sample, design, and procedure

The study was approved by the ethics committee of the Faculty of Arts and Social Sciences at the University of Zurich (22.10.5) and preregistered at https://osf.io/qt7zy/?view_only=9f0ae5877024489daca434b08aba7c3c. We recruited 300 participants through Prolific, stratified by age, gender, and ethnicity. Participants made decisions in a 2 (situation: competitive vs cooperative) × 2 (political ideology: leftist vs rightist) within-subjects design. They first read and were asked if they agreed to the consent form. Participants were then informed that they would make decisions in two tasks. Then, they were asked a question about their political identification (leftist or rightist). In the decision-making phase, they read instructions of the dictator game and of the tournament game (the order of the two games was randomized). In the dictator game, participants made three decisions in the role of allocators. In the tournament game, participants performed a task where they needed to estimate the number of dots across 11 trials. After that, we administered one block of questionnaires and socio-demographics. At the end of the survey, participants were shortly debriefed about the scope of the study. After data collection, we randomly selected one game, and paid participants for the decisions made in that game.

*Dictator game***.** Participants made three decisions in the dictator game, one with an ingroup member, one with an outgroup member, and one with an unidentified stranger. In the dictator game, there was an allocator and a recipient. Allocators could freely distribute an amount of 10 MU between themselves and the recipients. Participants made decisions as allocators knowing that each MU was worth 1 minute of average wage in the UK (0.20 GBP). Participants were informed that if the game was selected for payment, they would be matched with another participant, randomly assigned the role of allocator or recipient and paid accordingly.

Tournament game. In the tournament game, participants performed a dot estimation task. This task required participants to estimate several images that differ in the number of yellow dots. Each image contained 100 dots and is shown for 2 seconds. They were asked to estimate the number of dots across 11 trials. Before

starting with the 11 trials, participants were informed that they could decide to be paid according to two options: in the first option they competed with people that identified themselves as leftists, knowing that if they selected this option, they would be randomly paired with a person from the leftists group, and that they would receive 1 MU each time they are closer to the correct answer than this person. The second option was the same, except that in this case they would be compared with people that identified themselves as rightists.

5.2. Results

In line with the trust game results from Study 4, participants in this complementary study gave more resources to ingroup members (M = 3.86, SD = 1.90), compared to outgroup members and strangers (M = 3.24, SD = 1.93, $t(299)$= 8.45, $p < .001$). Parochial generosity also correlated with social identification (b = 0.254, $p < .001$).

Regarding self-selection into the tournament, we find that 39% of the participants decided to engage in the tournament with ingroup members and 61% of the participants decided to engage in the tournament with outgroup members ($X_{\text{squared}} = 14.52$, $p < .001$). This higher fraction of people selecting into a tournament with outgroup members was not predicted by social identification (generalized linear model of ingroup vs outgroup frequency predicted by social identification: b = -0.005, p = 0.96). In fact, we found that this pattern was mostly explained by stereotypes of intelligence and skills – when excluding participants that did not select based on such stereotypes, the difference between self-selection into ingroup or outgroup competition indeed disappears (when participants did not consider the outgroup unintelligent: $X_{\text{squared}} = 1.07$, p = 0.30; when they did not consider the outgroup unskilled: $X_{\text{squared}} = 0.04$, p = 0.85). Accordingly, the preference for competing against an outgroup member likely results from a stereotypical belief that political outgroups are unintelligent and unskilled, rather than from ingroup favouritism in competition. Indeed, across the 11 trials we also checked whether people were more accurate in their dot estimation when competing with ingroup vs outgroup: we found no significant differences in competitive performance: $t(262.97) = 0.899$, $p = .37$. Furthermore, in previous studies that implemented an option where participants were indifferent to ingroup and outgroup members, such option was chosen by the majority of the participants (*52*). We conclude that these results support neither the nasty neighbour effect nor ingroup favouritism.

Relation between parochial generosity and self-selection into tournament. As a further test, we also checked whether ingroup favouritism in generosity could explain or was associated with self-selection in competition with outgroup members. If that was the case, we should see that people opting to compete with outgroup members were also the ones that give more to ingroup members in the dictator game. This was, however, not the case. Selecting to compete with an ingroup vs outgroup individuals did not predict

differences in generosity between ingroup and outgroup members: $t(249.86) = -1.19$, $p = 0.24$). This provides additional support that self-selecting into competition was not driven by ingroup favouritism, and that competitive motivation can co-exist with ingroup favouritism.

Other Supplementary Material for this manuscript include the following:

Data S1. Dataset and code to reproduce the results reported in the manuscript.

REFERENCES AND NOTES

1. N. R. Buchan, G. Grimalda, R. Wilson, M. Brewer, E. Fatas, M. Foddy, Globalization and human cooperation. *Proc. Natl. Acad. Sci. U.S.A.* **106**, 4138–4142 (2009).

2. D. Balliet, J. Wu, C. K. W. De Dreu, Ingroup favoritism in cooperation: A meta-analysis. *Psychol. Bull.* **140**, 1556–1581 (2014).

3. G. Charness, Y. Chen, Social identity, group behavior, and teams. *Annu. Rev. Econom.* **12**, 691–713 (2020).

4. N. Masuda, F. Fu, Evolutionary models of in-group favoritism. *F1000Prime Rep.* **7**, 27 (2015).

5. N. Halevy, G. Bornstein, L. Sagiv, "In-group love" and "out-group hate" as motives for individual participation in intergroup conflict: A new game paradigm. *Psychol. Sci.* **19**, 405–411 (2008).

6. C. K. W. De Dreu, J. Gross, A. Fariña, Y. Ma, Group cooperation, carrying-capacity stress, and intergroup conflict. *Trends Cogn. Sci.* **24**, 760–776 (2020).

7. A. R. Dorrough, A. Glöckner, Multinational investigation of crosssocietal cooperation. *Proc. Natl. Acad. Sci. U.S.A.* **113**, 10836–10841 (2016).

8. C. K. W. De Dreu, A. Fariña, J. Gross, A. Romano, Prosociality as a foundation for intergroup conflict. *Curr. Opin. Psychol.* **44**, 112–116 (2022).

9. J. Gross, Z. Z. Méder, C. K. W. De Dreu, A. Romano, W. E. Molenmaker, L. C. Hoenig, The evolution of universal cooperation. *Sci. Adv.* **9**, eadd8289 (2023).

10. M. B. Brewer, The psychology of prejudice: Ingroup love and outgroup hate? *J. Soc. Issues* **55**, 429–444 (1999).

11. G. Spadaro, J. H. Liu, R. J. Zhang, H. Gil de Zúñiga, D. Balliet, Identity and institutions as foundations of ingroup favoritism: An investigation across 17 countries. *Soc. Psychol. Personal. Sci.***15**, 592–602 (2023).

12. A. Romano, M. Sutter, J. H. Liu, T. Yamagishi, D. Balliet, National parochialism is ubiquitous across 42 nations around the world. *Nat. Commun.* **12**, 4456 (2021).

13. C. K. W. De Dreu, Z. Triki, Intergroup conflict: Origins, dynamics and consequences across taxa. *Philos. Trans. R. Soc. B* **377**, 20210134 (2022).

14. S. R. T. Lemoine, L. Samuni, C. Crockford, R. M. Wittig, Parochial cooperation in wild chimpanzees: A model to explain the evolution of parochial altruism. *Philos. Trans. R. Soc. B* **377**, 20210149 (2022).

15. A. Romano, D. Balliet, T. Yamagishi, J. H. Liu, Parochial trust and cooperation across 17 societies. *Proc. Natl. Acad. Sci. U.S.A.* **114**, 12702–12707 (2017).

16. O. Weisel, R. Zultan, Perceptions of conflict: Parochial cooperation and outgroup spite revisited. *Organ. Behav. Hum. Decis. Process.* **167**, 57–71 (2021).

17. H. Aaldering, F. S. Ten Velden, G. A. van Kleef, C. K. W. De Dreu, Parochial cooperation in nested intergroup dilemmas is reduced when it harms out-groups. *J. Pers. Soc. Psychol.* **114**, 909–923 (2018).

18. H. Bernhard, U. Fischbacher, E. Fehr, Parochial altruism in humans. *Nature* **442**, 912–915 (2006).

19. A. C. Pisor, C. T. Ross, Parochial altruism: What it is and why it varies. *Evol. Hum. Behav.* **45**, 2–12 (2024).

20. C. K. W. De Dreu, L. L. Greer, M. J. J. Handgraaf, S. Shalvi, G. A. Van Kleef, M. Baas, F. S. Ten Velden, E. Van Dijk, S. W. W. Feith, The neuropeptide oxytocin regulates parochial altruism in intergroup conflict among humans. *Science* **328**, 1408–1411 (2010).

21. S. Fiedler, M. D. Hellmann, A. R. Dorrough, A. Glöckner, Cross-national ingroup favoritism in prosocial behavior: Evidence from Latin and North America. *Judgm. Decis. Mak.* **13**, 42–60 (2018).

22. S. Bowles, Being human: Conflict: Altruism's midwife. *Nature* **456**, 326–327 (2008).

23. J. K. Choi, S. Bowles, The coevolution of parochial altruism and war. *Science* **318**, 636–640 (2007).

24. N. R. Buchan, M. B. Brewer, G. Grimalda, R. K. Wilson, E. Fatas, M. Foddy, Global social identity and global cooperation. *Psychol. Sci.* **22**, 821–828 (2011).

25. K. Ito, M. Doebeli, The joint evolution of cooperation and competition. *J. Theor. Biol.* **480**, 1–12 (2019).

26. X. Basurto, E. Blanco, M. Nenadovic, B. Vollan, Integrating simultaneous prosocial and antisocial behavior into theories of collective action. *Sci. Adv.* **2**, e150122 (2016).

27. J. A. Lacomba, F. Lagos, E. Reuben, F. van Winden, On the escalation and de-escalation of conflict. *Games Econ. Behav.* **86**, 40–57 (2014).

28. W. Van Den Bos, J. Li, T. Lau, E. Maskin, J. D. Cohen, P. R. Montague, S. M. Mcclure, The value of victory: Social origins of the winner's curse in common value auctions. *Judgm. Decis. Mak.* **3**, 483–492 (2008).

29. D. Rand, M. A. Nowak, Human cooperation. *Trends Cogn. Sci.* **17**, 413–425 (2013).

30. R. Kranton, M. Pease, S. Sanders, S. Huettel, Deconstructing bias in social preferences reveals groupy and not-groupy behavior. *Proc. Natl. Acad. Sci. U.S.A.* **117**, 21185–21193 (2020).

31. D. Simunovic, N. Mifune, T. Yamagishi, Preemptive strike: An experimental study of fear-based aggression. *J. Exp. Soc. Psychol.* **49**, 1120–1123 (2013).

32. M. Dyble, The evolution of altruism through war is highly sensitive to population structure and to civilian and fighter mortality. *Proc. Natl. Acad. Sci. U.S.A.* **118**, e2011142118 (2021).

33. T. Lane, Discrimination in the laboratory: A meta-analysis of economics experiments. *Eur. Econ. Rev.* **90**, 375–402 (2016).

34. J. D. Fearon, D. D. Laitin, Ethnicity, insurgency, and civil war. *Am. Polit. Sci. Rev.* **97**, 75–90 (2003).

35. C. K. W. De Dreu, J. Gross, Revisiting the form and function of conflict: Neurobiological, psychological and cultural mechanisms for attack and defense within and between groups. *Behav. Brain Sci.* **42**, e116 (2019).

36. R. W. Wranghama, Two types of aggression in human evolution. *Proc. Natl. Acad. Sci. U.S.A.* **115**, 245–253 (2018).

37. C. K. W. De Dreu, M. Giacomantonio, M. R. Giffin, G. Vecchiato, Psychological constraints on aggressive predation in economic contests. *J. Exp. Psychol. Gen.* **148**, 1767–1781 (2019).

38. E. Dechenaux, D. Kovenock, R. M. Sheremeta, A survey of experimental research on contests, all-pay auctions and tournaments. *Exp. Econ.* **18**, 609–669 (2015).

39. T. Yamagishi, N. Mifune, Parochial altruism: Does it explain modern human group psychology? *Curr. Opin. Psychol.* **7**, 39–43 (2016).

40. D. J. Hruschka, J. Henrich, Economic and evolutionary hypotheses for cross-population variation in parochialism. *Front. Hum. Neurosci.* **7**, 559 (2013).

41. T. Mayer, S. Zignago, "CEPII - Notes on CEPII's distances measures: The GeoDist database," Working paper, CEPII, December 2011. www.cepii.fr/CEPII/en/publications/wp/abstract.asp?NoDoc=3877.

42. M. Muthukrishna, A. V. Bell, J. Henrich, C. M. Curtin, A. Gedranovich, J. McInerney, B. Thue, Beyond Western, Educated, Industrial, Rich, and Democratic (WEIRD) psychology: Measuring and mapping scales of cultural and psychological distance. *Psychol. Sci.* **31**, 678–701 (2020).

43. C. Boone, Land conflict and distributive politics in Kenya. *Afr. Stud. Rev.* **55**, 75–103 (2012).

44. Saferworld, “Why peace remains elusive as Kenya prepares for the 2022 general elections” (Saferworld, 2022); www.saferworld.org.uk/long-reads/why-peace-remains-elusive-as-kenya-prepares-for-the-2022-general-elections.

45. C. Christensen, A. N. Radford, Dear enemies or nasty neighbors? Causes and consequences of variation in the responses of group-living species to territorial intrusions. *Behav. Ecol.* **29**, 1004–1013 (2018).

46. C. A. Müller, M. B. Manser, “Nasty neighbours” rather than “dear enemies” in a social carnivore. *Proc. Biol. Sci.* **274**, 959–965 (2007).

47. J. Yoon, T. S. Sillett, S. A. Morrison, C. K. Ghalambor, Breeding density, not life history, predicts interpopulation differences in territorial aggression in a passerine bird. *Anim. Behav.* **84**, 515–521 (2012).

48. A. Benten, H. B. Cross, H. V. Tinnesand, A. Zedrosser, F. Rosell, Distant neighbours: Friends or foes? Eurasian beavers show context-dependent responses to simulated intruders. *Behav. Ecol. Sociobiol.* **74**, 17 (2020).

49. X. Niu, Z. Guan, W. Ning, X. Li, G. Sun, Q. Ni, G. Liu, X. Jiang, Experimental evidence for nasty neighbour effect in western black crested gibbons (*Nomascus concolor*). *Behav. Ecol. Sociobiol.* **77**, 33 (2023).

50. M. Decellieres, K. Zuberbühler, J. León, Habitat-dependent intergroup hostility in Diana monkeys, *Cercopithecus diana*. *Anim. Behav.* **178**, 95–104 (2021).

51. C. Camerer, *Behavioral Game Theory* (Russell Sage Foundation, 2003).

52. N. Mifune, D. Simunovic, T. Yamagishi, Intergroup biases in fear-induced aggression. *Front. Psychol.* **8**, 49 (2017).

53. R. B. Bouncken, J. Gast, S. Kraus, M. Bogers, Coopetition: A systematic review, synthesis, and future research directions. *Rev. Manag. Sci.* **9**, 577–601 (2015).

54. E. Zwirner, N. Raihani, Neighbourhood wealth, not urbanicity, predicts prosociality towards strangers. *Proc. R. Soc. B* **287**, 20201359 (2020).

55. E. Fehr, S. Gächter, Altruistic punishment in humans. *Nature* **415**, 137–140 (2002).

56. W. Xie, B. Ho, S. Meier, X. Zhou, Rank reversal aversion inhibits redistribution across societies. *Nat. Hum. Behav.*. **1**, 0142 (2017).

57. A. F. M. Martinangeli, L. Windsteiger, Last word not yet spoken: A reinvestigation of last place aversion with aversion to rank reversals. *Exp. Econ.* **24**, 800–820 (2021).

58. B. Herrmann, C. Thöni, S. Gächter, Antisocial punishment across societies. *Science* **319**, 1362–1367 (2008).

59. N. Mifune, Y. Hizen, Y. Kamijo, Y. Okano, Preemptive striking in individual and group conflict. *PLOS ONE* **11**, e0154859 (2016).

60. Y. Jing, P. H. Gries, Y. Li, A. W. Stivers, N. Mifune, D. M. Kuhlman, L. Bai, War or peace? How the subjective perception of great power interdependence shapes preemptive defensive aggression. *Front. Psychol.* **8**, 864 (2017).

61. F. Cornaglia, M. Drouvelis, P. Masella, Competition and the role of group identity. *J. Econ. Behav. Organ.* **162**, 136–145 (2019).

62. S. S. Liu, M. W. Morris, T. Talhelm, Q. Yang, Ingroup vigilance in collectivistic cultures. *Proc. Natl. Acad. Sci. U.S.A.* **116**, 14538–14546 (2019).

63. D. P. Fry, P. Söderberg, Lethal aggression in mobile forager bands and implications for the origins of war. *Science* **341**, 270–273 (2013).

64. P. S. Newey, S. K. A. Robson, R. H. Crozier, Weaver ants Oecophylla smaragdina encounter nasty neighbors rather than dear enemies. *Ecology* **91**, 2366–2372 (2010).

65. R. Böhm, H. Rusch, J. Baron, The psychology of intergroup conflict: A review of theories and measures. *J. Econ. Behav. Organ.* **178**, 947–962 (2020).

66. L. M. W. Li, G. Adams, T. Kurtiş, T. Hamamura, Beware of friends: The cultural psychology of relational mobility and cautious intimacy. *Asian J. Soc. Psychol.* **18**, 124–133 (2015).

67. G. Adams, The cultural grounding of personal relationship: Enemyship in North American and West African worlds. *J. Pers. Soc. Psychol.* **88**, 948–968 (2005).

68. T. Yamagishi, T. Kiyonari, The group as the container of generalized reciprocity. *Soc. Psychol. Q.* **63**, 116–132 (2000).

69. H. Tajfel, M. G. Billig, R. P. Bundy, C. Flament, Social categorization and intergroup behaviour. *Eur. J. Soc. Psychol.* **1**, 149–178 (1971).

70. H. K. Reeve, B. Ho, The emergence of a superorganism through intergroup competition. *Proc. Natl. Acad. Sci. U.S.A.* **104**, 9736–9740 (2007).

71. P. Richerson, R. Baldini, A. V. Bell, K. Demps, K. Frost, V. Hillis, S. Mathew, E. K. Newton, N. Naar, L. Newson, C. Ross, P. E. Smaldino, T. M. Waring, M. Zefferman, Cultural group selection plays an essential role in explaining human cooperation: A sketch of the evidence. *Behav. Brain Sci.* **39**, e30 (2016).

72. W. D. Hamilton, The genetical evolution of social behaviour. I. *J. Theor. Biol.* **7**, 1–16 (1964).

73. P. D. Taylor, Altruism in viscous populations - an inclusive fitness model. *Evol. Ecol.* **6**, 352–356 (1992).

74. P. D. Taylor, A. J. Irwin, Overlapping generations can promote altruistic behaviors. *Evolution* **54**, 1135–1141 (2000).

75. L. Lehmann, N. Perrin, F. O. Rousset, Population demography and the evolution of helping behaviors. *Evolution* **60**, 1137–1151 (2006).

76. A. Gardner, S. A. West, Demography, altruism, and the benefits of budding. *J. Evol. Biol.* **19**, 1707–1716 (2006).

77. G. Wild, A. D. Fernandes, Investment in the public good through conditional phenotypes of large effect. *J. Evol. Biol.* **22**, 927–941 (2009).

78. A. Romano, J. Gross, C. K. W. De Dreu, Conflict misperceptions between citizens and foreigners across the globe. *PNAS Nexus* **1**, pgac267 (2022).

79. D. Bates, M. Mächler, B. M. Bolker, S. C. Walker, Fitting linear mixed-effects models using lme4. *J. Stat. Softw.* **67**, 1–48 (2015).

80. A. Falk, A. Becker, T. Dohmen, B. Enke, D. Huffman, U. Sunde, Global evidence on economic preferences. *Q. J. Econ.* **133**, 1645–1692 (2018).

81. T. Postmes, S. A. Haslam, L. Jans, A single-item measure of social identification: Reliability, validity, and utility. *Br. J. Soc. Psychol.* **52**, 597–617 (2013).

82. Y. Long, Y. Li, The longitudinal association between social status insecurity and relational aggression: Moderation effects of social cognition about relational aggression. *Aggress. Behav.* **46**, 84–96 (2020).

83. C. K. W. De Dreu, H. S. Scholte, F. A. A. M. Van Winden, K. R. Ridderinkhof, Oxytocin tempers calculated greed but not impulsive defense in predator-prey contests. *Soc. Cogn. Affect. Neurosci.* **10**, 721–728 (2015).

84. Z. Z. Méder, C. K. W. De Dreu, J. Gross, Equilibria of attacker-defender games. arXiv:2202.10072 [econ.TH] (21 February 2022).

85. J. Berg, J. Dickhaut, K. McCabe, Trust, reciprocity, and social history. *Games Econ. Behav.* **10**, 122–142 (1995).

86. D. Tingley, T. Yamamoto, K. Hirose, L. Keele, K. Imai, "mediation: R package for causal mediation analysis" (UCLA Statistics/American Statistical Association, 2014); https://dspace.mit.edu/handle/1721.1/91154.

87. M. Niederle, L. Vesterlund, Do women shy away from competition? Do men compete too much? *Q. J. Econ.* **122**, 1067–1101 (2007).
